# Eps8 controls Src- and FAK-dependent phenotypes in squamous carcinoma cells

**DOI:** 10.1242/jcs.157560

**Published:** 2014-12-15

**Authors:** Christina Schoenherr, Bryan Serrels, Charlotte Proby, Debbie L. Cunningham, Jane E. Findlay, George S. Baillie, John K. Heath, Margaret C. Frame

**Affiliations:** 1 Edinburgh Cancer Research Centre, Institute of Genetics and Molecular Medicine, University of Edinburgh, Crewe Road South, EH4 2XR Edinburgh, UK; 2 Division of Cancer Research, Ninewells Hospital and Medical School, University of Dundee, Dundee DD1 9SY, UK; 3 Cancer Research UK Growth Factor Signalling Group, School of Biosciences, College of Life and Environmental Sciences, University of Birmingham, Birmingham B15 2TT, UK; 4 Institute of Cardiovascular and Medical Sciences, College of Medical, Veterinary and Life Sciences, University of Glasgow, Glasgow G12 8QQ, UK

**Keywords:** Eps8, FAK, Invasion, Autophagy, Src, Actin

## Abstract

Eps8 is an actin regulatory scaffold protein whose expression is increased in squamous cell carcinoma (SCC) cells. It forms a complex with both focal adhesion kinase (FAK, also known as PTK2) and Src in SCC cells derived from skin carcinomas induced by administration of the chemical DMBA followed by TPA (the DMBA/TPA model). Here, we describe two new roles for Eps8. Firstly, it controls the spatial distribution of active Src in a FAK-dependent manner. Specifically, Eps8 participates in, and regulates, a biochemical complex with Src and drives trafficking of Src to autophagic structures that SCC cells use to cope with high levels of active Src when FAK is absent. Secondly, when FAK is expressed in SCC cells, thereby meaning active Src becomes tethered at focal adhesion complexes, Eps8 is also recruited to focal adhesions and is required for FAK-dependent polarization and invasion. Therefore, Eps8 is a crucial mediator of Src- and FAK-regulated processes; it participates in specific biochemical complexes and promotes actin re-arrangements that determine the spatial localization of Src, and modulates the functions of Src and FAK during invasive migration.

## INTRODUCTION

Epidermal growth factor receptor kinase substrate 8 (Eps8) was originally identified as a substrate for receptor tyrosine kinases, including epidermal growth factor receptor (EGFR), fibroblast growth factor receptor (FGFR), platelet-derived growth factor receptor (PDGFR) and ErbB-2 (Fazioli et al., 1993). Through direct binding of receptor tyrosine kinases, Eps8 is involved in Rac signaling and receptor endocytosis (Auciello et al., 2013; Lanzetti et al., 2000). Additionally, Eps8 is a direct binding partner and phosphorylation target of the Src non-receptor tyrosine kinase (Cunningham et al., 2013; Maa et al., 1999).

Eps8 exists in two isoforms with molecular masses of 68 kDa and 97 kDa that are proposed to be alternative splice isoforms or proteolytic products (Fazioli et al., 1993; Maa et al., 1999), although their individual functions remain poorly characterized with most studies referring mainly to the 97-kDa isoform. Eps8 consists of an N-terminal phosphotyrosine-binding (PTB) domain, an SH3 domain and a C-terminal effector domain, through which Eps8 is thought to direct actin regulatory functions, such as capping barbed ends and promoting bundling (Disanza et al., 2004; Disanza et al., 2006; Hertzog et al., 2010). Eps8 binds the adaptor protein Abi-1 through its SH3 domain, releasing autoinhibitory binding within Eps8 (Disanza et al., 2004) and promoting actin capping. In addition, the role of Eps8 on stimulation of the Rac signaling pathway is mediated by the formation of a tri-complex consisting of Eps8, Abi-1 and Sos-1 (Innocenti et al., 2003; Scita et al., 1999).

Biologically, Eps8 regulates cell protrusions (Welsch et al., 2007), migration of dendritic cells (Frittoli et al., 2011), and morphogenesis of intestinal cells and microvilli (Croce et al., 2004; Zwaenepoel et al., 2012). In addition, Eps8 regulates stereocilia function and length, and *Eps8*^−/−^ mice are deaf (Manor et al., 2011; Olt et al., 2014; Zampini et al., 2011). This role of Eps8 in stereocilia function is supported by the description of a rare human germinal Eps8 mutation leading to hearing impairment (Behlouli et al., 2014). Eps8 expression is increased in several different cancer types, such as pancreatic cancer and oral squamous cell carcinoma (Chu et al., 2012; Welsch et al., 2007; Yap et al., 2009). It is proposed to play a role in squamous carcinogenesis and elevated Eps8 expression correlates with poor survival of cervical cancer patients (Chen et al., 2008; Wang et al., 2009). The 97-kDa isoform of Eps8 has been variously linked to proliferation, migration and oncogenic transformation (Leu et al., 2004; Liu et al., 2010; Maa et al., 2001; Maa et al., 2007), implying that the roles of Eps8 role in cancer cell phenotypes is complex and might be dependent on context.

Here, we have addressed the role of Eps8 in control of FAK- (also known as PTK2) and Src-dependent phenotypes in a well-defined cancer cell system. This is because we previously found that: (1) Eps8 is hugely upregulated in squamous cell carcinoma (SCC) cells derived from skin carcinomas induced by administration of the chemicals 7,12-dimethylbenz(a)anthracene (DMBA) followed by 12-*O*-tetradecanoylphorbol-13-acetate) (TPA) [the DMBA/TPA model; driven by mutated oncogenic H-Ras (Quintanilla et al., 1986)] when compared to normal keratinocytes and in human SCCs, and (2) it associates with both FAK and active Src in SCC cells. In previous work, we found that expression of FAK was also enhanced during SCC progression (Agochiya et al., 1999), and that genetic deletion of *fak* suppressed tumorigenesis, particularly progression to malignant carcinoma in the DMBA/TPA model (McLean et al., 2004). Moreover, we have shown that FAK-dependent cancer cell phenotypes are associated with polarization and directional migration that require the scaffolding function of FAK, including the binding to actin regulatory proteins, such as Arp3 and RACK1 (Serrels et al., 2010; Serrels et al., 2007). In FAK-deficient SCC cells, we have also shown that active Src (and other FAK-binding tyrosine kinases such as Ret) are targeted away from peripheral focal adhesions into actin-associated intracellular puncta containing autophagy regulators, permitting FAK-deficient cells to use non-classical autophagic mechanisms to cope with high levels of unregulated active Src in the absence of its ‘tethering partner’ at focal adhesions (Sandilands et al., 2012a; Sandilands et al., 2012b).

We describe two new roles for Eps8: (1) when FAK is absent, Eps8 participates in a biochemical complex that controls the targeting of Src to autophagic structures, probably through effects on associated actin re-arrangements, and (2) when FAK is present (and co-upregulated with Eps8), Eps8 and FAK form a complex that is required for Src- and FAK-dependent cancer cell invasion *in vitro*.

## RESULTS

### Eps8 expression is increased in SCC cells

**Fig. 1.**
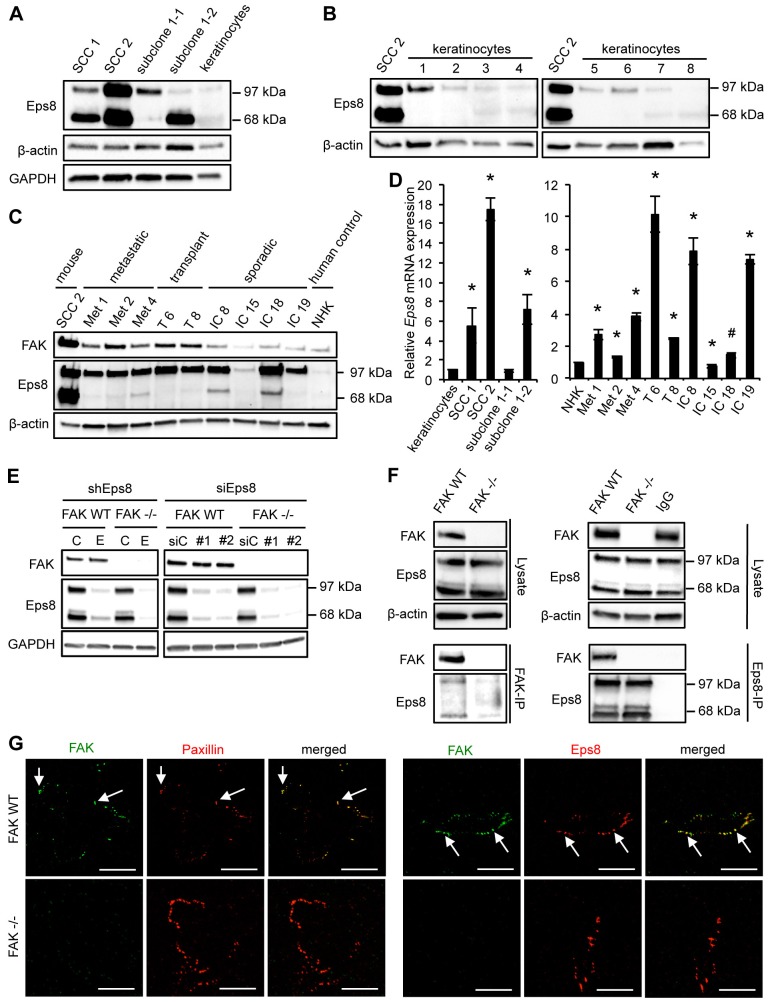
**Eps8 expression is upregulated in SCCs and interacts with FAK.** (A) Primary keratinocytes were isolated from mouse tails, and Eps8 expression was compared to a number of SCC lines by western blotting using anti-Eps8 antibody. Anti-β-actin (middle panel) and anti-GAPDH (lower panel) antibodies were used as loading controls. (B) Eps8 expression was compared between SCC cell line 2 (SCC 2) and independent keratinocyte cultures by western blotting using anti-Eps8 antibody (upper panel). Anti-β-actin was used as a loading control (lower panel). (C) Eps8 expression was compared between SCC 2, human SCC cell lines and a human NHK control cell line by western blotting using anti-Eps8 antibody (middle panel). Met, metastatic SCC lines; T, SCC lines from patients who had previously received organ transplants; IC, SCC lines from patients with sporadic primary SCC. FAK expression was analyzed employing anti-FAK antibody (upper panel) and anti-β-actin was used as a loading control (lower panel). (D) Relative *Eps8* mRNA expression in mouse and human SCC cell lines was analyzed by qRT-PCR using the ΔΔCt method. GAPDH was used as a control for differences in cDNA input. Results are mean±s.d. *P<0.01; ^#^*P*<0.05 (Student's *t*-test). (E) FAK expression upon knockdown of endogenous Eps8 by shRNA (shEps8 E lanes; C lanes show control shRNA) or two independent siRNAs (siEps8 #1 and #2; siC, control siRNA) was analyzed using anti-FAK antibody (upper panel). Eps8 knockdown was confirmed by using anti-Eps8 antibody (middle panel) and anti-GAPDH was used as a loading control (lower panel). (F) FAK or Eps8 were immunoprecipitated (IP) from FAK WT and FAK^−/−^ cell lysates using anti-FAK antibody conjugated to agarose (clone 4.47) (left panel) or anti-Eps8 antibody (right panel), followed by western blot analysis with anti-FAK and anti-Eps8 antibodies (lower panels). Eps8 immunoblotting detects both known isoforms with molecular masses of 97 kDa and 68 kDa. Anti-β-actin was used as a loading control. (G) Focal adhesions were isolated from FAK WT and FAK^−/−^ cells using hydrodynamic force. Left panels: focal adhesions (arrows) were stained with anti-FAK and anti-paxillin antibodies. Right panels: focal adhesions (solid arrows) were stained with anti-FAK and anti-Eps8. Scale bars: 20 µm.

We found that Eps8 expression (both isoforms) was elevated in SCC cells derived from the DMBA/TPA model of chemical carcinogenesis (SCC 1 and SCC 2 cells; Fig. 1A) when compared to primary keratinocytes isolated from mouse tails (as described in Materials and Methods). Two malignant SCC subclones, derived from one of the SCC cell lines (SCC 1; subclones 1-1 and 1-2) expressed much higher Eps8 levels than primary keratinocytes. Although the SCC 1 cell line expressed both isoforms of Eps8, the individual subclones predominantly expressed one or the other of the two isoforms; specifically, SCC 1-2 expressed the 68-kDa form, whereas SCC 1-1 expressed the 97-kDa form, although the functional significance of this difference is unknown. The SCC 2 cell line expressed both isoforms to a similar extent (Fig. 1A). To rule out variability between keratinocytes, we also compared Eps8 expression in SCC 2 to eight different isolates of primary keratinocytes (keratinocytes 1–8). The SCC 2 cell line expressed substantially higher Eps8 when compared to all primary keratinocyte isolates (Fig. 1B). As Eps8 is reported to be increased in oral SCC (Chu et al., 2012; Yap et al., 2009), we next investigated whether Eps8 expression levels were increased in human cutaneous squamous cell carcinoma cells. We tested three cell lines from patients with metastatic SCC (Fig. 1C,D, labeled Met), two from patients who had previously received organ transplants (Fig. 1C,D, labeled T) and so were immune suppressed, and four from patients with sporadic primary SCC (Fig. 1C,D, labeled IC). Normal human keratinocytes (NHKs) served as normal counterparts for the human cell lines (Watt et al., 2011). In eight out of nine human cell lines, Eps8 expression was enhanced when compared to NHKs (Fig. 1C). All human SCC cell lines predominantly expressed the 97-kDa form of Eps8. Enhanced Eps8 expression can largely be explained by increased transcription: three out of four mouse and eight out of nine human SCC cell lines showed increased *Eps8* mRNA compared to primary keratinocytes or NHKs, respectively. One cell line (IC 15) had slightly decreased *Eps8* mRNA expression levels, and that was reflected in no visible increase in the steady-state level of Eps8 protein (Fig. 1D). We also confirmed that, although there was general co-upregulation of Eps8 and FAK, their elevated expression was not inter-dependent as was proposed previously (Maa et al., 2007). Reduction of Eps8 by stable or transient knockdown had no effect on FAK expression (Fig. 1E), and genetic deletion of FAK did not influence Eps8 levels in SCC cells (Fig. 1E,F).

These data show that Eps8 expression is generally elevated in SCC cells compared to normal keratinocytes and is independently co-overexpressed with FAK, which is also increased in DMBA/TPA-induced murine SCC cells and in human SCC cells (Agochiya et al., 1999; Frame et al., 2010; McLean et al., 2004).

### Eps8 interacts with FAK in SCC cells at focal adhesions

We identified Eps8 as a potential binding partner of FAK in a proteomic screen (data not shown). This interaction was confirmed by co-immunoprecipitation (using either anti-Eps8 or anti-FAK as primary antibody) in SCC cells that were wild-type (WT) for FAK. These are cells in which WT FAK is re-expressed to endogenous levels in FAK^−/−^ SCC cells from which the gene encoding endogenous FAK had undergone prior deletion by Cre-mediated recombination of a floxed-*fak* allele [as we have described previously (Sandilands et al., 2012a; Serrels et al., 2010)]. Confirming FAK deficiency, the 125-kDa species identified as FAK was absent from Eps8 immunoprecipitations of lysates of FAK^−/−^ SCC cells (Fig. 1F). We noted that both Eps8 isoforms (97 kDa and 68 kDa) interacted with FAK. To investigate whether Eps8 and FAK were both present at focal adhesions, we isolated these structures from SCC FAK WT and FAK^−/−^ cells using hydrodynamic force (as described in the Materials and Methods). Residual adhesion structures were confirmed by staining with the focal adhesion protein paxillin (Fig. 1G, left panels, arrows), and FAK and Eps8 were both present with 100% colocalization (Fig. 1G, right panels, arrows; supplementary material Fig. S1A). To examine whether or not Eps8 was present at nascent focal adhesions, FAK WT and FAK^−/−^ SCC cells were treated with blebbistatin – an inhibitor of cellular contractility (Straight et al., 2003; Zhang and Rao, 2005) – which reduced the size of focal adhesions as expected (supplementary material Fig. S1B). In blebbistatin-treated SCC FAK WT cells, Eps8 appeared to be colocalized with FAK (supplementary material Fig. S1B, left panel) and active Src (phosphorylated at Y416; p-Src, supplementary material Fig. S1B, right panel) at small, presumed nascent, adhesions. The adhesion colocalization was confirmed by total internal reflection fluorescence (TIRF) microscopy (supplementary material Fig. S1C–E). Taken together, these data show that FAK and Eps8 form a complex, and colocalize, at focal adhesion sites in SCC cells.

### Eps8 is required for FAK-dependent actin-associated cancer phenotypes

**Fig. 2.**
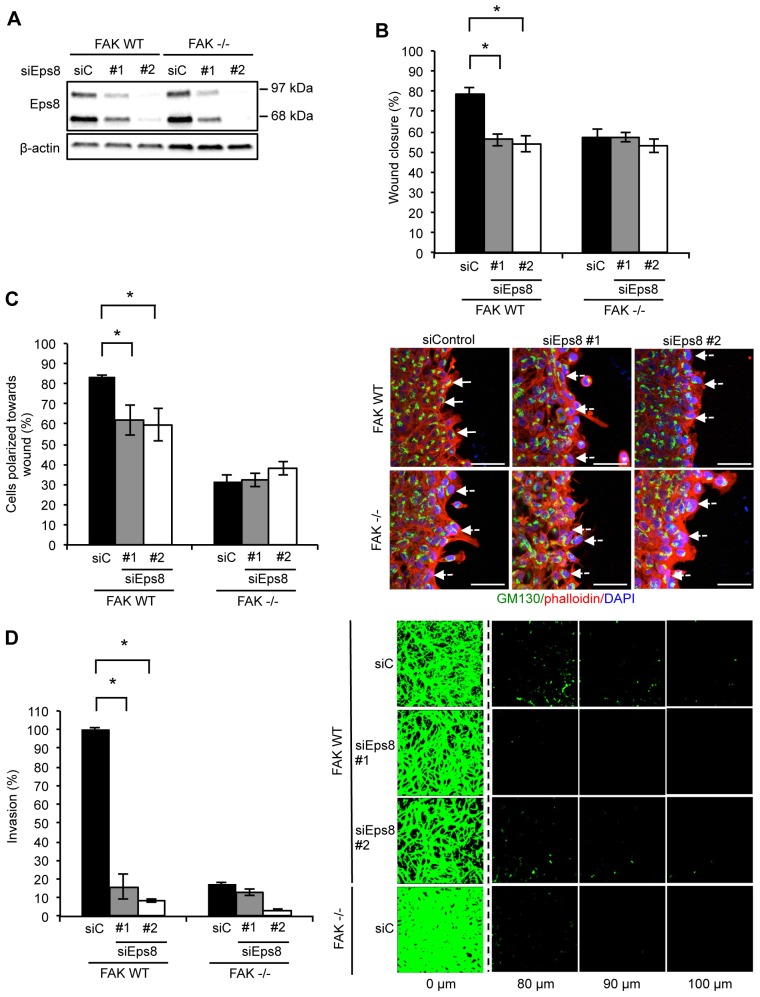
**Eps8 regulates polarized cell migration and invasion.** FAK WT and FAK^−/−^ cells were transiently transfected with two independent Eps8 siRNAs (siEps8 #1 and #2; siC, control siRNA) and (A) lysed 48 h post transfection and Eps8 expression determined by western blotting using anti-Eps8 antibody. Anti-β-actin was used as a loading control. (B) Confluent monolayers of cells plated on fibronectin were wounded using a pipette tip. Wound closure was quantified at 15 h post wounding. Results are mean±s.e.m. *P<0.01 (Student's *t*-test). (C) Confluent monolayers of cells plated on fibronectin were wounded using a pipette tip, fixed 3 h later and stained with anti-GM130 antibody (to label the Golgi) and TRITC–phalloidin. Polarization was scored using the orientation of the Golgi towards the wound edge. Solid arrows indicate polarized cells. Dashed arrows indicate unpolarized cells. Scale bars: 20 µm. Results are mean±s.d. *P<0.001 (Student's *t*-test). (D) Cells were seeded on growth-factor-reduced Matrigel in serum-free conditions. Invasion towards a serum gradient (the distance of the horizontal *z*-section from the top of Matrigel is given) was visualized after 72 h by staining the cells with calcein. Results are mean±s.e.m. *P<0.001 (Student's *t*-test). Quantification is representative of at least three independent experiments in all cases.

We next investigated the role of Eps8 in FAK-mediated cancer-associated phenotypes, such as migration, polarization and invasion through Matrigel. Two independent small interfering RNAs (siRNAs) targeting Eps8 (denoted siEps8 #1 and #2) were used to robustly knockdown endogenous Eps8 expression (Fig. 2A). We analyzed wound closure by assessing the migration of cells plated on fibronectin, having first ruled out that Eps8 depletion did not obviously affect cell proliferation in two-dimensional culture (supplementary material Fig. S2A,B). SCC cells required FAK for optimal wound closure, and this was also suppressed by Eps8 knockdown in FAK WT cells (Fig. 2B). Wound closure of FAK-deficient cells was not further suppressed by Eps8 knockdown, suggesting that their effects are linked.

Next, we addressed the role of Eps8 in cell polarization towards a wound in a confluent monolayer, and in permitting cancer cell invasion into, and through, Matrigel in response to serum used as a chemoattractant. Like FAK, Eps8 was required for cell polarization on fibronectin, as measured by the orientation of the Golgi (stained with anti-GM130) towards the wound (Fig. 2C) (Serrels et al., 2010). As we described previously, FAK deficiency inhibits invasion through Matrigel, whereas FAK-expressing SCCs efficiently migrate through Matrigel (Serrels et al., 2012). Invasion was also strongly inhibited (more than fivefold) upon depletion of endogenous Eps8 in SCC cells (Fig. 2D). Similar results were obtained with stable knockdown of Eps8 by short hairpin RNA (shRNA) (supplementary material Fig. S2F–H). Thus, FAK and Eps8 are both absolutely required for polarization and invasive migration.

**Fig. 3.**
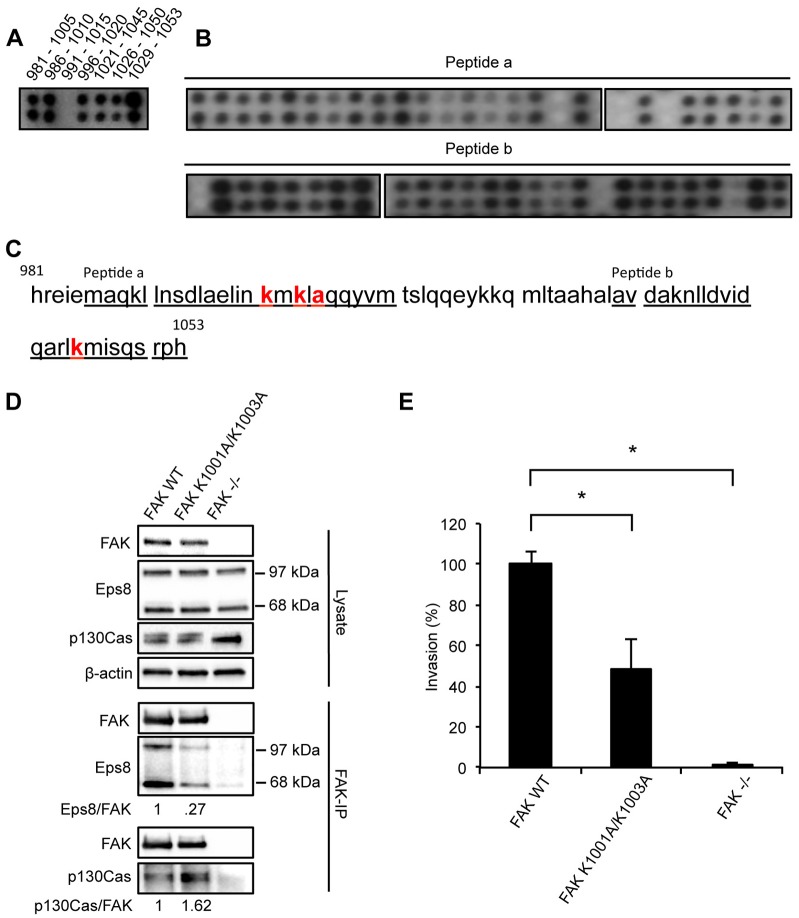
**Eps8 binds to the C-terminus of FAK.** (A–C) Identification of the Eps8-binding sequence in FAK. (A) Overlapping 25-mer peptides spanning the C-terminus of FAK (amino acids 981–1053) were spotted onto nitrocellulose, incubated with recombinant Eps8 and probed with anti-Eps8 antibody. (B) The core binding sequences in peptide a (top panel) and peptide b (bottom panel) were identified by alanine scanning where one amino acid was mutated at a time. (C) The sequences of the 25-mer peptides (peptide a and peptide b) used for the alanine scanning are underlined and the identified core binding sites is marked in bold red. (D) FAK was immunoprecipitated (IP) from FAK WT, FAK K1001A/K1003A and FAK^−/−^ cell lysates using anti-FAK antibody conjugated to agarose (clone 4.47), followed by western blot analysis with anti-FAK, anti-Eps8 and anti-p130Cas antibodies (lower panels). Eps8 immunoblotting detects both known isoforms with molecular masses of 97 kDa and 68 kDa. Anti-β-actin antibody was used as a loading control. (E) Invasion assay. SCC FAK WT, FAK K1001A/K1003A and FAK^−/−^ cells were seeded on growth factor reduced Matrigel in serum-free conditions. Invasion towards serum gradient was visualized after 72 h by staining the cells with calcein. Results are mean±s.e.m. *P≤0.01 (Student's *t*-test). Quantification is representative of three independent experiments.

In order to confirm whether the interaction between FAK and Eps8, and the biological consequences of their depletion, were dependent on their direct interaction, we mapped the Eps8-binding site in FAK by peptide array binding analysis (Fig. 3; we described the method previously in Serrels et al., 2010; Serrels et al., 2007). Briefly, overlapping 25-mer peptides spanning the FAK sequence were spotted onto nitrocellulose, followed by incubation with recombinant Eps8. The peptide array was washed extensively, incubated with anti-Eps8 antibody and then subjected to immunoblotting. Eps8 bound to the focal-adhesion-targeting (FAT) domain of FAK, spanning the region of the amino acids 981–1053 (Fig. 3A). For the identification of the amino acids in FAK responsible for binding Eps8, overlapping 25-mers had one amino acid mutated at a time to alanine. We identified that peptide ‘a’ (amino acids 986–1010) contained the binding amino acids K1001, K1003 and A1005, whereas peptide ‘b’ (amino acids 1029–1053) contained the binding amino acid K1045 (Fig. 3B,C). We made several combinations of mutations, and showed that one of these, FAK K1001A/K1003A, in which the lysine residues at positions 1001 and 1003 were changed to alanine residues, caused reduced binding of both isoforms of Eps8 to FAK (Fig. 3D). However, the interaction between FAK and p130Cas (also known as BCAR1), which binds FAK at different residues in the proline-rich regions of FAK [P715, P718, P878 and P881 (Harte et al., 1996)] was unaffected. When binding of Eps8 to FAK was suppressed by expression of the FAK K1001A/K1003A mutant in otherwise FAK-deficient cells, we found that SCC invasion through Matrigel was significantly impaired (Fig. 3E). This is consistent with an important role for the FAK–Eps8 complex in mediating cancer cell invasion as measured *in vitro*.

### Eps8 interacts with active Src at adhesions and autophagosomes

**Fig. 4.**
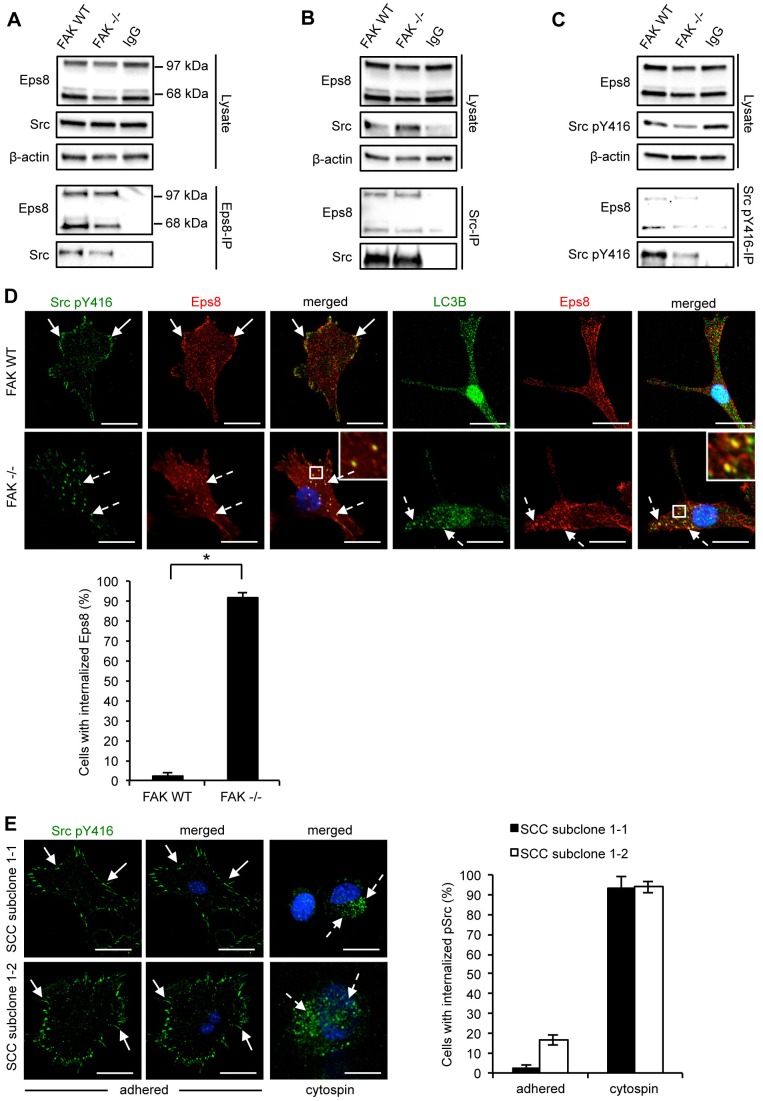
**Src–Eps8 complexes localize to autophagosomes in the absence of FAK.** Eps8, Src or Src pY416 were immunoprecipitated (IP) from FAK WT or FAK^−/−^ cell lysates using (A) anti-Eps8, (B) anti-Src or (C) anti-Src pY416 antibodies followed by western blotting analysis with antibodies as indicated (lower panels). Anti-β-actin antibody was used as a loading control. (D) FAK WT and FAK^−/−^ cells were grown on glass coverslips, fixed and stained using anti-Src pY416 and anti-Eps8 (left hand panels) or anti-LC3B and anti-Eps8 (right hand panels). Solid arrows indicate focal adhesions (upper panels) and dashed arrows indicate internalized active Src or Eps8 (lower panels). Scale bars: 20 µm. Insets show a magnified view of the boxed area. A quantification is shown below the images. Results are mean±s.d. *P<0.001 (Student's *t*-test). (E) SCC subclone 1-1 and subclone 1-2 cells were suspended in PBS for 1 h and cytospins were prepared and stained for Src pY416 and DAPI. Solid arrows indicate focal adhesions (upper panels) and dashed arrows indicate internalized active Src or Eps8 (lower panels). Results are mean±s.d. The quantifications are representative of 100 cells from three independent experiments. Scale bars: 20 µm.

Next, we addressed whether Eps8 also interacted with Src in SCC cells. Both Eps8 isoforms bound to active Src (phosphorylated at Y416; p-Src) in FAK WT and FAK^−/−^ cells, as shown by reciprocal co-immunoprecipitations (Fig. 4A–C). Hence, the complex between Eps8 and active Src did not depend on the Eps8 interaction with FAK. Furthermore, whereas Eps8 and p-Src localized in peripheral adhesion structures in FAK-expressing SCC cells, Eps8 was also colocalized with p-Src in intracellular puncta in FAK-deficient SCC cells (Fig. 4D). We recently reported that active Src is trafficked from peripheral adhesions into intracellular puncta that also contain autophagy proteins in FAK-deficient cells, as a consequence of improper scaffolding and tethering of highly active Src at focal adhesions (Sandilands et al., 2012a). Hence, in SCC cells that do not express FAK, Eps8 is co-targeted with active Src to autophagosomal puncta (Fig. 4D, left-hand image panels, dashed arrows). This was confirmed by co-staining with the autophagosomal marker LC3B (also known as MAP1LC3B) (Fig. 4D, right-hand image panels). Blebbistatin treatment blocked the trafficking of active Src from focal adhesions to autophagosomes (supplementary material Fig. S1B, right panel), suggesting that actomyosin contractility was required. As SCC cells express both the 68-kDa and 97-kDa Eps8 isoforms, we were able to address whether these differentially regulated the trafficking of active Src to autophagosomes. To do this, we tested the two subclones that expressed only one isoform, namely SCC subclone 1-1 (97 kDa isoform) and SCC subclone 1-2 (68 kDa isoform). Given that these subclones expressed FAK, we induced autophagosomal trafficking of Src by suspending the cells in PBS prior to cytospinning on to glass slides [as described previously (Sandilands et al., 2012a)]. In both SCC subclones, p-Src was present in intracellular puncta upon loss of cell–substrate adhesion, demonstrating that either isoform of Eps8 was sufficient to permit this intracellular trafficking of active Src (Fig. 4E).

We also examined whether tyrosine phosphorylation of Eps8 mediated by Src family kinases might be linked to its subcellular localization. We found that the tyrosine kinase inhibitor Dasatinib [which effectively inhibits the activities of Src family kinases (Lombardo et al., 2004)] suppressed Eps8 tyrosine phosphorylation (supplementary material Fig. S3A) and the internalization of Eps8 to intracellular puncta upon FAK deletion, whereas the number of LC3B-staining puncta remained unaltered (supplementary material Fig. S3B). These results suggest that tyrosine phosphorylation of Eps8 correlates with its co-recruitment with Src into autophagosomes, but not to general autophagy in SCC cells.

### Eps8 is required for efficient localization of active Src to autophagosomes

**Fig. 5.**
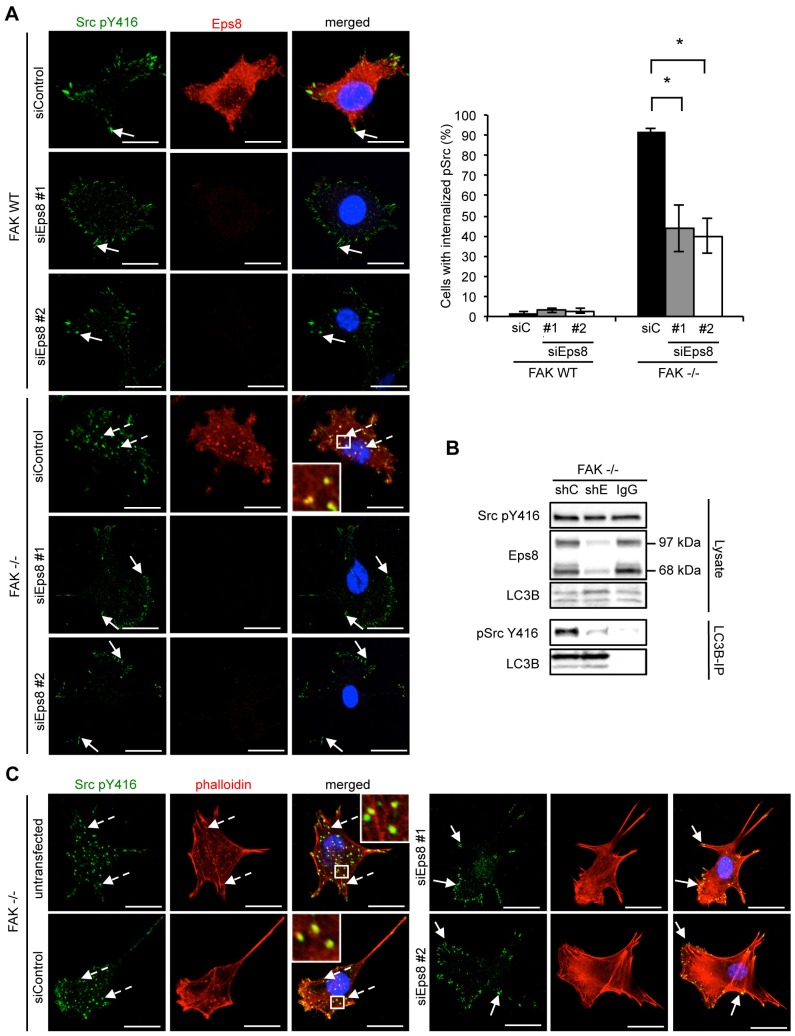
**Eps8 is required for active Src localization to autophagosomes.** (A) FAK WT and FAK^−/−^ cells were transiently transfected with Eps8 siRNA (siEps8 #1 and #2; siControl, control siRNA), fixed and stained with anti-Eps8 and anti-Src pY416 antibodies. The quantification is representative of three independent experiments. Solid arrows indicate focal adhesions. Dashed arrows indicate active Src in autophagosomes. Scale bars: 20 µm. Results are mean±s.d. *P<0.01 (Student's *t*-test). (B) FAK^−/−^ SCC cells were infected with either Eps8 (shE) or non-targeting (shC) shRNA and LC3B was immunoprecipitated (IP) from cell lysates. Samples were subjected to western blot analysis using anti-Eps8 and anti-Src pY416 antibodies. (C) FAK^−/−^ cells were transiently transfected with two independent Eps8 siRNAs, fixed and stained with anti-Src pY416 antibody and TRITC–phalloidin. Solid arrows indicate active Src localization at focal adhesions. Dashed arrows indicate active Src in intracellular puncta. Scale bars: 20 µm. Insets show a magnified view of the boxed area.

We next investigated whether Eps8 plays a key role in the trafficking of active Src to autophagosomes or whether it was co-trafficked as a ‘passenger’. We used two independent siRNAs to efficiently suppress expression of endogenous Eps8 as judged by immunofluorescence and immunoblotting (Fig. 1E; Fig. 2A; Fig. 5A). Knockdown of Eps8 in FAK WT SCC cells had no effect on the localization of active Src, which remained at focal adhesions (Fig. 5A, top panel, solid arrows). However, in FAK^−/−^ SCC cells, Eps8 knockdown significantly reduced the number of Src-positive intracellular puncta (Fig. 5A, bottom panels, dashed arrows). Active Src was now predominantly re-localized to focal adhesions as indicated by co-staining with paxillin (Fig. 5A, bottom panel, solid arrows and supplementary material Fig. S3C). Similar results were obtained by shRNA-mediated stable knockdown of Eps8 (supplementary material Fig. S3D,E). Furthermore, we found that the biochemical complex between active Src and LC3B in SCC FAK^−/−^ cells was reduced upon stable knockdown of Eps8, providing biochemical evidence that Eps8 function is required to induce the binding of Src to LC3B during autophagic targeting of Src. Eps8 did not affect general autophagic flux as judged by immunoblotting for LC3B (Fig. 5B), including after chloroquine treatment (supplementary material Fig. S4A,B). These results indicate that Eps8 is required for the selective trafficking of active Src to autophagosomes.

### Knockdown of Eps8 results in loss of actin changes associated with Src

We previously reported that tyrosine-kinase-containing autophagosomes are aligned with actin filaments, and colocalize with what appears in the confocal microscope as actin ‘patches’ or ‘nodules’ [Fig. 5C, dashed arrows (Sandilands et al., 2012b)]. As Eps8 is a known actin regulator, we found that the actin patches were no longer visible, as judged by phalloidin staining, when we induced transient knockdown of Eps8 by two independent siRNAs (Fig. 5C, solid arrows) and compared to control (untransfected and transfected with non-targeting siRNA) FAK^−/−^ SCC cells, in which internalized active Src colocalized with actin patches (Fig. 5C, dashed arrows). This demonstrates that Eps8 controls actin re-arrangements that are associated with Src-containing autophagosomes, but does not affect general autophagosomes as judged by the continued presence of LC3B-positive puncta upon Eps8 knockdown (supplementary material Fig. S4C).

**Fig. 6.**
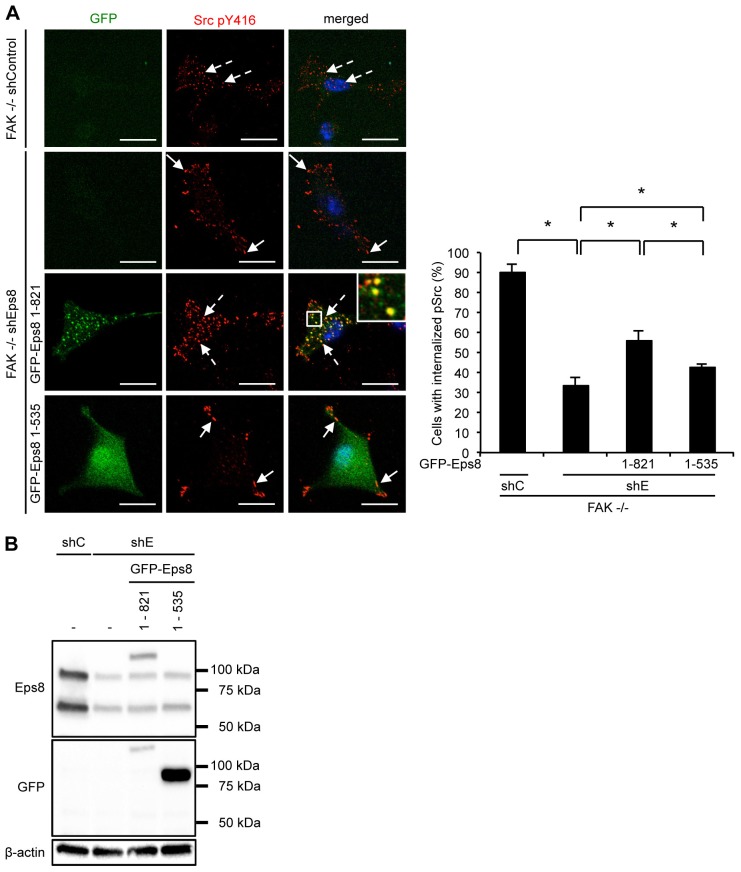
**The actin binding region of Eps8 mediates Src trafficking to autophagosomes.** SCC FAK^−/−^ cells, infected either with Eps8 (shE) or non-targeting (shC) shRNA were transiently transfected with either full-length Eps8 (GFP–Eps8 1–821) or with Eps8 lacking both the SH3 domain and the C-terminal actin binding region (GFP–Eps8 1–535). (A) Cells were fixed and stained with anti-Src pY416 antibody. The quantification is representative of five independent experiments. Scale bars: 20 µm. Results are mean±s.d. *P<0.01 (Student's *t*-test). (B) Cells were lysed and lysates were analyzed by western blotting analysis with the indicated antibodies. Immunoblotting with β-actin served as a loading control.

To test this further, we set out to investigate whether trafficking of Src to autophagosomes was dependent on the known actin regulatory function of Eps8, mediated by its actin-binding domains. SCC FAK^−/−^ cells in which Eps8 had been stably knocked down using shRNA were reconstituted with either full length Eps8 (GFP–Eps8 1–821) or Eps8 lacking the SH3 and the actin-binding domains (GFP–Eps8 1–535) (Welsch et al., 2010; Fig. 6). Full-length GFP–Eps8 localized to intracellular puncta and restored the localization of active Src to these in a significant number of cells, despite restitution of the GFP–Eps8 fusion protein to only low level when compared to endogenous Eps8 (Fig. 6A,B). In contrast, the Eps8 mutant lacking the SH3 and actin-binding domain displayed diffuse cytoplasmic localization, and was inefficient in restoring active Src localization to autophagic puncta, despite substantial overexpression when compared with GFP–Eps8 1–821 (Fig. 6A; note the C-terminal Eps8 epitope recognized by the Eps8 antibody was lost from the GFP–Eps8 1–535 (Fig. 6B, upper panel), and we detected the endogenous fusion protein with anti-GFP antibody (Fig. 6B; lower panel). These data imply that the trafficking of active Src to autophagosomes is controlled by the actin regulatory function of Eps8, consistent with loss of rearranged actin when Eps8 is knocked down (Fig. 5).

Overall, our data indicate a key role for Eps8 as a component of Src and FAK biochemical complexes that control the FAK-dependent actin-associated cancer phenotypes of polarization and invasive migration. However, although Eps8 is clearly an accessory protein for FAK at focal adhesions, it is also an important mediator of the spatial targeting of active Src from focal adhesions to autophagosomes upon FAK deletion or induction of adhesion stress. This identifies Eps8 as a mediator of integrin effector signaling and FAK-dependent downstream phenotypes in SCC cancer cells, likely by controlling the actin organization in the proximity of Src–Eps8 or FAK–Eps8 complexes.

## DISCUSSION

Eps8 is a molecular scaffold that regulates actin by assembling the complexes that control filament capping and bundling (Croce et al., 2004; Disanza et al., 2004; Frittoli et al., 2011; Hertzog et al., 2010). Through multi-protein complexes and signaling to the Rho GTPases Rac and Cdc42, Eps8 also controls the generation of membrane ruffles and filopodia, respectively (Disanza et al., 2006; Goicoechea et al., 2006; Innocenti et al., 2003). A number of studies have suggested that Eps8 expression is altered in cancer (for examples, see Maa et al., 2007; Welsch et al., 2007), and that it has putative roles in tumor cell proliferation and migration. Its precise role in cancer initiation or progression in mice has not been elucidated, largely because it shares redundant biological functions with other family members, namely Eps8L1, Eps8L2 and Eps8L3 (Fazioli et al., 1993). This has meant that deletion of the gene encoding Eps8 has no effect on murine embryonic development (Scita et al., 1999), whereas in *Caenorhabditis elegans*, where there is only one Eps8 ortholog, there is a clear developmental phenotype associated with impaired apical morphogenesis in the developing intestine. This is a result of an actin barbed-end capping dysfunction (Croce et al., 2004).

We studied Eps8 in mouse and human SCCs by using cell lines derived from: (1) DMBA/TPA (driven by mutated oncogenic H-Ras) tumors in mice (that also harbored a floxed *fak* allele) (McLean et al., 2004), and (2) patients presenting with either spontaneous SCCs, metastases or transplantation-induced SCCs, the latter of which were likely as a result of prolonged immune suppression (Watt et al., 2011). We found unequivocally that Eps8 gene product(s) are elevated in mouse SCC cell lines when compared to normal keratinocytes, and in patient-derived carcinomas, transplantation-induced SCCs and metastases. In mouse SCC cell lines, expression of either one or both of the prominent isoforms is increased. In patient-derived SCC cells, it is predominantly the slower-migrating 97-kDa isoform that is increased. The significance of selection of one or other for over-representation is not known, nor is the difference between mouse and human, although overexpression of the 97-kDa isoform of Eps8 has recognized oncogenic transforming properties (Maa et al., 2001). In keeping with our previous data (Agochiya et al., 1999; McLean et al., 2004), FAK expression was also elevated in mouse and human SCCs, particularly in transplant-induced carcinomas and metastases.

We identified Eps8 in an interaction proteomics screen for SCC-relevant FAK-binding partners, and observed co-immunoprecipitation. Although it has been reported that Eps8 controls FAK expression (Maa et al., 2007), we did not find any reciprocal inter-dependent regulation in SCC cells; FAK deletion did not influence Eps8 expression and Eps8 depletion did not repress FAK expression. Furthermore, in the human SCC cells lines IC 18 and IC 19, there was evidence of increased Eps8 expression without elevated FAK. Hence, we conclude Eps8 and FAK are molecular scaffolds whose expression levels are generally co-elevated in SCC cells, although their expression does not seem to be inter-dependent. However, Eps8 and FAK bind to one another directly and are likely part of larger multi-protein scaffolding complexes in cancer cells.

Eps8 depletion had similar effects on cancer-associated phenotypes to those seen upon deletion of FAK in SCC cells (Serrels et al., 2010), including impaired polarization and invasive migration, with there were no visible effects on random cell migration (supplementary material Fig. S2C–E). Furthermore, Eps8 depletion did not cause further impairment over that caused by loss of FAK alone, suggesting that Eps8 and FAK are promoting their cancer-associated processes by the same molecular pathway. This is supported by the fact that impairment of FAK binding to Eps8 inhibits invasion, implying that the FAK–Eps8 complex is important in cancer cell phenotypes. The effect of this complex is probably mediated through actin-regulatory scaffolding complexes at specific subcellular locale, most likely at integrin-associated focal adhesions, where both FAK and Eps8 reside in SCC cells. We conclude that although Eps8 shares overlapping functions with other family members during mammalian development, its role in cancer cell polarization and invasive migration cannot apparently be replaced by other Eps8 family members. Although it has been reported that Eps8 regulates colon cancer growth (Chen et al., 2008; Maa et al., 2007), we did not find proliferation to be dependent on Eps8 in SCCs from mouse or human tumors.

We recently described that highly active Src is removed from focal adhesions and transported into intracellular puncta when FAK is absent from SCC cells (Sandilands et al., 2012a). Essentially, Src [and other highly active FAK-binding tyrosine kinases, including Ret (Sandilands et al., 2012b)], are targeted from focal adhesions into intracellular puncta that contain multiple autophagy proteins, and active Src is restored at the cell periphery upon suppression of autophagy (Sandilands et al., 2012a; Sandilands et al., 2012b). We had noticed that phosphorylated Ret present in the same intracellular puncta showed a striking colocalization with actin-rich patches. These appeared to be aligned with bundled actin filaments that were tethered into focal adhesions (Sandilands et al., 2012b). However, up to this point we had not identified any actin regulatory proteins that were co-recruited to the Src- and Ret-containing autophagosomes, or that were responsible for mediating actin reorganization leading to formation of the actin-rich patches. In the present work, we found that Eps8 was a FAK-binding protein that was co-recruited with p-Src into autophagic intracellular puncta, and that siRNA- or shRNA-mediated depletion of Eps8 was essential for the efficient intracellular targeting of Src and the formation of a Src–LC3B complex when FAK was absent. When Eps8 was depleted, the polymerized actin-rich patches were no longer visible by phalloidin staining. This identifies a new type of actin structure that is regulated by Eps8, and which is linked to the trafficking of active Src from focal adhesions to intracellular autophagic puncta. Furthermore, both Eps8 isoforms were able to mediate trafficking of Src to intracellular puncta, suggesting that the C-terminal actin-binding effector domain, which is present in both Eps8 isoforms, is required. In keeping with this, overexpression of an Eps8 mutant lacking both the SH3 domain and the actin-binding effector region was unable to rescue the trafficking of active Src to autophagosomes. Eps8 has previously been found in lysosomes and is subject to chaperone-mediated autophagy in cancer cells (Welsch et al., 2010), suggesting that its cellular levels also need to be tightly regulated by lysosomal degradation pathways.

In summary, Eps8 is independently co-upregulated with FAK during the development of SCC both in human and mouse. Eps8 and FAK participate in a complex at focal adhesions that promotes actin-associated cancer phenotypes, including direction sensing and invasive migration. FAK is required for optimal targeting of Eps8 to, or its retention at, focal adhesion structures, and Eps8 binds to the focal-adhesion-targeting sequences of FAK. However, when FAK is absent, Eps8 is instead co-recruited with active Src to intracellular puncta that contain autophagy proteins, which we have previously shown SCC cancer cells use to deal with highly active Src that is not tethered to FAK. Our work identifies a new role for the actin regulator Eps8 in the intracellular targeting of active Src, most likely via the regulation of actin patches associated with autophagosomes. This provides another example of a role for Eps8 as a key mediator of the fate of intracellular kinases, adding to its role in signaling and trafficking of receptor tyrosine kinases, like EGFR and FGFR (Auciello et al., 2013; Fazioli et al., 1993).

## MATERIALS AND METHODS

### Antibodies, inhibitors and DNA constructs

Antibodies used were as follows: anti-Eps8, anti-paxillin, anti-GM130 and anti-p130Cas antibodies (BD Transduction Laboratories, NJ), anti-FAK, anti-pSrc Y416, anti-Src (clone 36D10), anti-LC3B, anti-GAPDH, anti-pPaxillin Y118 and anti-β-actin antibodies (Cell Signaling Technologies, Danvers, MA), as well as anti-FAK antibody conjugated to agarose (clone 4.47) (Millipore, Billerica, MA) and anti-LC3B for immunoprecipitations (MBL, Woburn, MA). The anti-TagCGYFP antibody was purchased from Evrogen (Cambridge, UK). TRITC–phalloidin was purchased from Life Technologies (Paisley, UK). Horseradish-peroxidase-conjugated secondary antibodies against rabbit or mouse IgG were purchased from Cell Signaling Technologies. Dasatinib was obtained from Bristol Myers Squibb (Princeton, NJ). The pEGFP-Eps8 1–821 and pEGFP-Eps8 1–535 constructs were a generous gift from Giorgio Scita (FIRC Institute of Molecular Oncology, Milan, Italy).

### Generation of FAK mutant constructs

FAK mutants were generated by site directed mutagenesis using PFU Ultra Hotstart DNA polymerase (Stratagene, Amsterdam, The Netherlands) and the following primers (mutated base pairs are underlined): K1001A/K1003A (forward 5′-CTGAGCTCATTAACGCGATGGCGCTGGCCCAGCAGTAC- 3′, reverse 5′- GTACTGCTGGGCCAGCGCCATCGCGTTAATGAGCTCAG- 3′). After DpnI digestion for 1 h at 37°C chemically competent TOP10 bacteria were transformed.

### Cell culture and transfection

FAK-deficient SCC cell lines were generated as described previously (Serrels et al., 2010). SCCs were maintained in Glasgow minimal essential medium (MEM) containing 10% fetal calf serum (FCS), 2 mM L-glutamine, non-essential amino acids, sodium pyruvate and MEM vitamins at 37°C and under 5% CO_2_. SCC FAK WT cells were maintained in 1 mg/ml hygromycin B. Cell lines with stable knockdown of Eps8 were maintained in 1 µg/ml puromycin. Primary keratinocytes were isolated from K14-Cre mouse tails as follows: skin was removed from the tails of adult mice and incubated in 4 mg/ml dispase in PBS for 2 h at 37°C. The epidermis was then isolated and cut into small pieces prior incubation in trypsin for 10 min at 37°C. DMEM with 20% FCS was added and the cells were filtered through a 70 µm nylon cell strainer. Cells were subsequently cultured in DMEM supplemented with 10% FCS and 2 mM L-glutamine. All animal experiments were performed according to approved guidelines.

Human SCC cell lines were described previously (Watt et al., 2011). Briefly, cells were cultured on Mitomycin C growth arrested 3T3 fibroblasts which were removed by differential trypsinization. Cells were maintained in RM+ medium [Dulbecco's modified Eagle's medium (DMEM) with Ham's F12, 10% FCS, 10 ng/ml EGF, 5 µg/ml insulin, 400 ng/ml hydrocortisone, 5 µg/ml transferrin, 8.4 ng/ml cholera toxin and 0.0177 nM lyothyronine</emph>]. All human samples were collected after informed, written consent and in accordance with the Helsinki guidelines.

### siRNAs and shRNAs

Eps8 siRNA (catalog number J-045154-11, 5′-ACGACUUUGUGGCGAGGAA-3′; A-045154-16, 5′- UUGGUAUAUGUAAUUUAUC-3′) or scrambled siRNA (catalog number D-001810-10) were purchased from Dharmacon, Loughborough, UK. FAK WT or FAK^−/−^ SCC cells were transiently transfected using HiPerFect (Qiagen, Manchester, UK), according to the manufacturer's protocol with a final concentration of 40 nM siRNA. Cells were analyzed at 48–96 h post transfection.

SCC FAK WT and FAK^−/−^ cells with stable Eps8 knockdown were generated using a lentiviral shRNA Eps8 construct (catalog number RMM4534; Thermo Scientific, Loughborough, UK). Briefly, HEK293 cells were transfected with Eps8 shRNA along with viral packaging constructs using Lipofectamine 2000 (Life Technologies, Paisley, UK). Virus-containing medium was collected twice over a 48-h period, filtered and diluted 1∶1 with fresh SCC complete growth medium supplemented with 5 µg/ml polybrene (Millipore, Billerica, MA, USA). Cells were selected for Eps8 shRNA expression using puromycin (Life Technologies, Paisley, UK) at a final concentration of 1 µg/ml.

### Mapping of the Eps8-binding site in FAK

The Eps8-binding site in FAK was identified using peptide arrays as published previously (Serrels et al., 2010; Serrels et al., 2007). Briefly, overlapping 25-mer peptides of FAK were spotted onto nitrocellulose and incubated with recombinant Eps8 (Abnova, Buckingham, UK). After extensive washes, the array was incubated with anti-Eps8 antibody and then subjected to western blotting. For the identification of core amino acids, overlapping 25-mer peptides with one amino acid mutated at a time were used.

### qRT-PCR

RNA from cells was isolated using the RNeasy Mini Kit (Qiagen, Manchester, UK). 500 ng of total RNA was reverse-transcribed using the SuperScript First-Strand cDNA synthesis kit (Life Technology, Paisley, UK). For the PCR amplification in a Step One Plus real-time PCR system (Life Technology, Paisley, UK), 25 ng cDNA were used in a total reaction mix of 20 µl containing 10 µl Sensi Fast SYBR Green Hi-Rox (Bioline, London, UK) as well as 400 nM forward and reverse primer. GAPDH was used to control for differences in cDNA input. The following primers were used: mouse Eps8 (forward 5′-GTCAACTCCTAATCACCAAGTAG-3′, reverse 5′-CTGTTCCTCGCCACAAAG-3′), mouse GAPDH (forward 5′-CGTCCCGTAGACAAAATGGT-3′, reverse 5′-TTGATGGCAACAATCTCCAC-3′), human Eps8 (forward 5′-GCCAACTTCTAATCGCCATA-3′, reverse 5′-TCACTGTTGTTCCTTGCTAC-3′) and human GAPDH (forward 5′-CCCCGGTTTCTATAAATTGAGC-3′, reverse 5′-CACCTTCCCCATGGTGTCT-3′). Relative expression was calculated according to the ΔΔCt quantification method. Each sample within an experiment was performed in triplicate and the experiment was carried out three times.

### Immunoblotting and immunoprecipitation

Cells were washed twice in ice-cold PBS and then lysed in RIPA buffer (50 mM Tris-HCl pH 8.0, 150 mM NaCl, 1% Triton X-100, 0.1% SDS and 0.5% sodium deoxycholate) or in NP40 lysis buffer (50 mM Tris-HCl pH 8.0, 150 mM NaCl, 0.5% NP40) supplemented with PhosStop and Complete Ultra Protease Inhibitor tablets (Roche, Welwyn Garden City, UK). Lysates were cleared by centrifugation at 9300 ***g*** for 15 min and analyzed by western blotting. Protein concentration was calculated using a BCA protein assay kit (Thermo Scientific, Loughborough, UK). For immunoprecipitation, 1 mg cell lysates were incubated with 2 µg of unconjugated or 10 µl of agarose-conjugated antibodies at 4°C overnight with agitation. Unconjugated antibody samples were incubated with 10 µl of Protein-A–agarose or Protein-G–agarose for 1 h at 4°C. Beads were washed three times in lysis buffer and once in 0.6 M LiCl, resuspended in 20 µl 2× sample buffer (130 mM Tris-HCl pH 6.8, 20% glycerol, 5% SDS, 8% β-mercaptoethanol and Bromphenol Blue) and heated for 5 min at 95°C. Samples were then subjected to SDS-PAGE analysis using the Bio-Rad TGX pre-cast gel system. Proteins were immunoblotted using the Bio-Rad Trans-blot Turbo transfer system, blocked in 5% BSA in TBS supplemented with 1% Tween-20 (TBST), and incubated with primary antibody overnight at 4°C. Blots were washed three times in TBST, incubated with horseradish-peroxidase-conjugated secondary antibody for 45 min at room temperature, washed as above, developed using Clarity Western ECL Substrate (Bio-Rad, Hemel Hempstead, UK) and imaged using a Bio-Rad ChemiDoc MP Imaging System (Bio-Rad, Hemel Hempstead, UK).

### Immunofluorescence microscopy and image analysis

Cells were grown on glass coverslips for 24 h and washed once in TBS prior to fixation (3.7% formaldehyde, 100 mM PIPES pH 6.8, 10 mM EGTA, 1 mM MgCl_2_ and 0.2% Triton X-100) for 10 min. Cells were subsequently washed twice in TBS supplemented with 0.1% Triton X-100 (TBStx) and blocked in TBStx block (TBStx supplemented with 3% BSA). Fixed cells were incubated with primary antibodies in TBStx block overnight at 4°C, followed by 3× 5 min washes in TBStx, and incubated with Alexa-Fluor-labeled secondary antibodies diluted 1∶200 in TBStx block (Life Technologies, Paisley, UK) and washed as before prior to being mounted in Vectashield-mounting medium containing DAPI (Vector Labs, Peterborough, UK). Cells were imaged using a FV1000 Confocal microscope (Olympus, Southend-on-Sea, UK). For the total internal reflection fluorescence (TIRF) microscopy, an inverted IX81 microscope (Olympus, Southend-on-Sea, UK) with a 150× 1.45 NA UAPON TIRF objective using 491 nm and 561 nm excitation lines was used. Colocalization was analyzed using the ImageJ plugin JaCoP (Bolte and Cordelières, 2006). For quantification of internalized p-Src or Eps8, 100 cells from three independent experiments were counted.

### Focal adhesion isolation

Focal adhesion isolation was performed following the protocol described in Kuo et al. (Kuo et al., 2012). Briefly, cells were rinsed with PBS and incubated with TEA buffer (0.2 M triethanolamine, pH 8.0) for 5 min. To apply hydrodynamic force, the cells were rinsed with PBS for 10 s using a Waterpik dental flosser set at 2 (Waterpik, Reigate, UK). After another wash with PBS, the remaining attached focal adhesions were fixed for immunofluorescence analysis.

### Cell migration assays

Cell migration was analyzed as described previously (Serrels et al., 2012). 10^6^ cells were grown on fibronectin-coated six-well plates for 15 h until cells were confluent. The cell monolayer was wounded with a pipette tip. Wound closure was monitored with an Olympus ScanR/CellR microscope (Olympus, Essex, UK). Images were taken every 15 min for 15 h and wound closure was analyzed using TScratch (Gebäck et al., 2009). The experiment was carried out three times.

### Cell polarization assays

Cell polarization assessing the orientation of the Golgi in wounded cell monolayers was examined as described previously (Serrels et al., 2010). Briefly, 3×10^6^ cells were plated on fibronectin-coated coverslips in 12-well plates for 3 h. The cell monolayer was wounded with a pipette tip, incubated in full SCC growth medium for 1.5 h and then fixed and stained with anti-GM130 antibody. The experiment was carried out three times.

### Invasion assays

Invasion was analyzed as described previously (Serrels et al., 2010). Briefly, growth factor reduced Matrigel (BD Biosciences, Oxford, UK) was diluted 1∶1 in cold PBS and allowed to set at 37°C in transwells. 2×10^4^ cells were seeded onto the underside of the transwell. After 4 h the transwells were washed in PBS and placed into serum free SCC growth medium. Full growth medium containing 10% FCS was added on top of the Matrigel. After 72 h cell invasion was assessed by staining with 5 µM calcein (Life Technologies, Paisley, UK) for 1 h. Horizontal *z* sections through the Matrigel were acquired at 10 µm intervals with an Olympus FV1000 confocal microscope. The images were evaluated using ImageJ software. The experiment was carried out three times.

## Supplementary Material

Supplementary Material

## References

[b1] Agochiya M. Brunton V. G. Owens D. W. Parkinson E. K. Paraskeva C. Keith W. N. Frame M. C. (1999). Increased dosage and amplification of the focal adhesion kinase gene in human cancer cells. Oncogene 18, 5646–5653 10.1038/sj.onc.1202957 10523844

[b2] Auciello G. Cunningham D. L. Tatar T. Heath J. K. Rappoport J. Z. (2013). Regulation of fibroblast growth factor receptor signalling and trafficking by Src and Eps8. J. Cell Sci. 126, 613–624 10.1242/jcs.116228 23203811 PMC3613183

[b3] Behlouli A. Bonnet C. Abdi S. Bouaita A. Lelli A. Hardelin J. P. Schietroma C. Rous Y. Louha M. Cheknane A. (2014). EPS8, encoding an actin-binding protein of cochlear hair cell stereocilia, is a new causal gene for autosomal recessive profound deafness. Orphanet J. Rare Dis. 9, 55 10.1186/1750-1172-9-55 24741995 PMC4022326

[b4] Bolte S. Cordelières F. P. (2006). A guided tour into subcellular colocalization analysis in light microscopy. J. Microsc. 224, 213–232 10.1111/j.1365-2818.2006.01706.x 17210054

[b5] Chen Y. J. Shen M. R. Chen Y. J. Maa M. C. Leu T. H. (2008). Eps8 decreases chemosensitivity and affects survival of cervical cancer patients. Mol. Cancer Ther. 7, 1376–1385 10.1158/1535-7163.MCT-07-2388 18566210

[b6] Chu P. Y. Liou J. H. Lin Y. M. Chen C. J. Chen M. K. Lin S. H. Yeh C. M. Wang H. K. Maa M. C. Leu T. H. (2012). Expression of Eps8 correlates with poor survival in oral squamous cell carcinoma. Asia Pac. J. Clin. Oncol. 8, e77–e81 10.1111/j.1743-7563.2011.01459.x 22897151

[b7] Croce A. Cassata G. Disanza A. Gagliani M. C. Tacchetti C. Malabarba M. G. Carlier M. F. Scita G. Baumeister R. Di Fiore P. P. (2004). A novel actin barbed-end-capping activity in EPS-8 regulates apical morphogenesis in intestinal cells of Caenorhabditis elegans. Nat. Cell Biol. 6, 1173–1179 10.1038/ncb1198 15558032

[b8] Cunningham D. L. Creese A. J. Auciello G. Sweet S. M. Tatar T. Rappoport J. Z. Grant M. M. Heath J. K. (2013). Novel binding partners and differentially regulated phosphorylation sites clarify Eps8 as a multi-functional adaptor. PLoS ONE 8, e61513 10.1371/journal.pone.0061513 23626693 PMC3634024

[b9] Disanza A. Carlier M. F. Stradal T. E. Didry D. Frittoli E. Confalonieri S. Croce A. Wehland J. Di Fiore P. P. Scita G. (2004). Eps8 controls actin-based motility by capping the barbed ends of actin filaments. Nat. Cell Biol. 6, 1180–1188 10.1038/ncb1199 15558031

[b10] Disanza A. Mantoani S. Hertzog M. Gerboth S. Frittoli E. Steffen A. Berhoerster K. Kreienkamp H. J. Milanesi F. Di Fiore P. P. (2006). Regulation of cell shape by Cdc42 is mediated by the synergic actin-bundling activity of the Eps8-IRSp53 complex. Nat. Cell Biol. 8, 1337–1347 10.1038/ncb1502 17115031

[b11] Fazioli F. Minichiello L. Matoska V. Castagnino P. Miki T. Wong W. T. Di Fiore P. P. (1993). Eps8, a substrate for the epidermal growth factor receptor kinase, enhances EGF-dependent mitogenic signals. EMBO J. 12, 3799–3808. 8404850 10.1002/j.1460-2075.1993.tb06058.x PMC413663

[b12] Frame M. C. Patel H. Serrels B. Lietha D. Eck M. J. (2010). The FERM domain: organizing the structure and function of FAK. Nat. Rev. Mol. Cell Biol. 11, 802–814 10.1038/nrm2996 20966971

[b13] Frittoli E. Matteoli G. Palamidessi A. Mazzini E. Maddaluno L. Disanza A. Yang C. Svitkina T. Rescigno M. Scita G. (2011). The signaling adaptor Eps8 is an essential actin capping protein for dendritic cell migration. Immunity 35, 388–399 10.1016/j.immuni.2011.07.007 21835647 PMC3424277

[b14] Gebäck T. Schulz M. M. Koumoutsakos P. Detmar M. (2009). TScratch: a novel and simple software tool for automated analysis of monolayer wound healing assays. Biotechniques 46, 265–274. 19450233 10.2144/000113083

[b15] Goicoechea S. Arneman D. Disanza A. Garcia-Mata R. Scita G. Otey C. A. (2006). Palladin binds to Eps8 and enhances the formation of dorsal ruffles and podosomes in vascular smooth muscle cells. J. Cell Sci. 119, 3316–3324 10.1242/jcs.03076 16868024

[b16] Harte M. T. Hildebrand J. D. Burnham M. R. Bouton A. H. Parsons J. T. (1996). p130Cas, a substrate associated with v-Src and v-Crk, localizes to focal adhesions and binds to focal adhesion kinase. J. Biol. Chem. 271, 13649–13655 10.1074/jbc.271.23.13649 8662921

[b17] Hertzog M. Milanesi F. Hazelwood L. Disanza A. Liu H. Perlade E. Malabarba M. G. Pasqualato S. Maiolica A. Confalonieri S. (2010). Molecular basis for the dual function of Eps8 on actin dynamics: bundling and capping. PLoS Biol. 8, e1000387 10.1371/journal.pbio.1000387 20532239 PMC2879411

[b18] Innocenti M. Frittoli E. Ponzanelli I. Falck J. R. Brachmann S. M. Di Fiore P. P. Scita G. (2003). Phosphoinositide 3-kinase activates Rac by entering in a complex with Eps8, Abi1, and Sos-1. J. Cell Biol. 160, 17–23 10.1083/jcb.200206079 12515821 PMC2172734

[b19] Kuo J. C. Han X. Yates J. R. III and Waterman C. M. (2012). Isolation of focal adhesion proteins for biochemical and proteomic analysis. Methods Mol. Biol. 757, 297–323 10.1007/978-1-61779-166-6_19 21909920 PMC4158431

[b20] Lanzetti L. Rybin V. Malabarba M. G. Christoforidis S. Scita G. Zerial M. Di Fiore P. P. (2000). The Eps8 protein coordinates EGF receptor signalling through Rac and trafficking through Rab5. Nature 408, 374–377 10.1038/35042605 11099046

[b21] Leu T. H. Yeh H. H. Huang C. C. Chuang Y. C. Su S. L. Maa M. C. (2004). Participation of p97Eps8 in Src-mediated transformation. J. Biol. Chem. 279, 9875–9881 10.1074/jbc.M309884200 14699156

[b22] Liu P. S. Jong T. H. Maa M. C. Leu T. H. (2010). The interplay between Eps8 and IRSp53 contributes to Src-mediated transformation. Oncogene 29, 3977–3989 10.1038/onc.2010.144 20418908

[b23] Lombardo L. J. Lee F. Y. Chen P. Norris D. Barrish J. C. Behnia K. Castaneda S. Cornelius L. A. Das J. Doweyko A. M. (2004). Discovery of N-(2-chloro-6-methyl- phenyl)-2-(6-(4-(2-hydroxyethyl)- piperazin-1-yl)-2-methylpyrimidin-4- ylamino)thiazole-5-carboxamide (BMS-354825), a dual Src/Abl kinase inhibitor with potent antitumor activity in preclinical assays. J. Med. Chem. 47, 6658–6661 10.1021/jm049486a 15615512

[b24] Maa M. C. Lai J. R. Lin R. W. Leu T. H. (1999). Enhancement of tyrosyl phosphorylation and protein expression of eps8 by v-Src. Biochim. Biophys. Acta 1450, 341–351 10.1016/S0167-4889(99)00069-5 10395945

[b25] Maa M. C. Hsieh C. Y. Leu T. H. (2001). Overexpression of p97Eps8 leads to cellular transformation: implication of pleckstrin homology domain in p97Eps8-mediated ERK activation. Oncogene 19, 106–112 10.1038/sj.onc.1204069 11244499

[b26] Maa M. C. Lee J. C. Chen Y. J. Chen Y. J. Lee Y. C. Wang S. T. Huang C. C. Chow N. H. Leu T. H. (2007). Eps8 facilitates cellular growth and motility of colon cancer cells by increasing the expression and activity of focal adhesion kinase. J. Biol. Chem. 282, 19399–19409 10.1074/jbc.M610280200 17496330

[b27] Manor U. Disanza A. Grati M. Andrade L. Lin H. Di Fiore P. P. Scita G. Kachar B. (2011). Regulation of stereocilia length by myosin XVa and whirlin depends on the actin-regulatory protein Eps8. Curr. Biol. 21, 167–172 10.1016/j.cub.2010.12.046 21236676 PMC3040242

[b28] McLean G. W. Komiyama N. H. Serrels B. Asano H. Reynolds L. Conti F. Hodivala-Dilke K. Metzger D. Chambon P. Grant S. G. (2004). Specific deletion of focal adhesion kinase suppresses tumor formation and blocks malignant progression. Genes Dev. 18, 2998–3003 10.1101/gad.316304 15601818 PMC535910

[b29] Olt J. Mburu P. Johnson S. L. Parker A. Kuhn S. Bowl M. Marcotti W. Brown S. D. (2014). The actin-binding proteins eps8 and gelsolin have complementary roles in regulating the growth and stability of mechanosensory hair bundles of mammalian cochlear outer hair cells. PLoS ONE 9, e87331 10.1371/journal.pone.0087331 24475274 PMC3903700

[b30] Quintanilla M. Brown K. Ramsden M. Balmain A. (1986). Carcinogen-specific mutation and amplification of Ha-ras during mouse skin carcinogenesis. Nature 322, 78–80 10.1038/322078a0 3014349

[b31] Sandilands E. Serrels B. McEwan D. G. Morton J. P. Macagno J. P. McLeod K. Stevens C. Brunton V. G. Langdon W. Y. Vidal M. (2012a). Autophagic targeting of Src promotes cancer cell survival following reduced FAK signalling. Nat. Cell Biol. 14, 51–60 10.1038/ncb2386 22138575

[b32] Sandilands E. Serrels B. Wilkinson S. Frame M. C. (2012b). Src-dependent autophagic degradation of Ret in FAK-signalling-defective cancer cells. EMBO Rep. 13, 733–740 10.1038/embor.2012.92 22732841 PMC3410392

[b33] Scita G. Nordstrom J. Carbone R. Tenca P. Giardina G. Gutkind S. Bjarnegård M. Betsholtz C. Di Fiore P. P. (1999). EPS8 and E3B1 transduce signals from Ras to Rac. Nature 401, 290–293 10.1038/45822 10499589

[b34] Serrels B. Serrels A. Brunton V. G. Holt M. McLean G. W. Gray C. H. Jones G. E. Frame M. C. (2007). Focal adhesion kinase controls actin assembly via a FERM-mediated interaction with the Arp2/3 complex. Nat. Cell Biol. 9, 1046–1056 10.1038/ncb1626 17721515

[b35] Serrels B. Sandilands E. Serrels A. Baillie G. Houslay M. D. Brunton V. G. Canel M. Machesky L. M. Anderson K. I. Frame M. C. (2010). A complex between FAK, RACK1, and PDE4D5 controls spreading initiation and cancer cell polarity. Curr. Biol. 20, 1086–1092 10.1016/j.cub.2010.04.042 20493699

[b36] Serrels A. McLeod K. Canel M. Kinnaird A. Graham K. Frame M. C. Brunton V. G. (2012). The role of focal adhesion kinase catalytic activity on the proliferation and migration of squamous cell carcinoma cells. Int. J. Cancer 131, 287–297 10.1002/ijc.26351 21823119

[b37] Straight A. F. Cheung A. Limouze J. Chen I. Westwood N. J. Sellers J. R. Mitchison T. J. (2003). Dissecting temporal and spatial control of cytokinesis with a myosin II Inhibitor. Science 299, 1743–1747 10.1126/science.1081412 12637748

[b38] Wang H. Patel V. Miyazaki H. Gutkind J. S. Yeudall W. A. (2009). Role for EPS8 in squamous carcinogenesis. Carcinogenesis 30, 165–174 10.1093/carcin/bgn252 19008210

[b39] Watt S. A. Pourreyron C. Purdie K. Hogan C. Cole C. L. Foster N. Pratt N. Bourdon J. C. Appleyard V. Murray K. (2011). Integrative mRNA profiling comparing cultured primary cells with clinical samples reveals PLK1 and C20orf20 as therapeutic targets in cutaneous squamous cell carcinoma. Oncogene 30, 4666–4677 10.1038/onc.2011.180 21602893 PMC3219832

[b40] Welsch T. Endlich K. Giese T. Büchler M. W. Schmidt J. (2007). Eps8 is increased in pancreatic cancer and required for dynamic actin-based cell protrusions and intercellular cytoskeletal organization. Cancer Lett. 255, 205–218 10.1016/j.canlet.2007.04.008 17537571

[b41] Welsch T. Younsi A. Disanza A. Rodriguez J. A. Cuervo A. M. Scita G. Schmidt J. (2010). Eps8 is recruited to lysosomes and subjected to chaperone-mediated autophagy in cancer cells. Exp. Cell Res. 316, 1914–1924 10.1016/j.yexcr.2010.02.020 20184880 PMC4304094

[b42] Yap L. F. Jenei V. Robinson C. M. Moutasim K. Benn T. M. Threadgold S. P. Lopes V. Wei W. Thomas G. J. Paterson I. C. (2009). Upregulation of Eps8 in oral squamous cell carcinoma promotes cell migration and invasion through integrin-dependent Rac1 activation. Oncogene 28, 2524–2534 10.1038/onc.2009.105 19448673

[b43] Zampini V. Rüttiger L. Johnson S. L. Franz C. Furness D. N. Waldhaus J. Xiong H. Hackney C. M. Holley M. C. Offenhauser N. (2011). Eps8 regulates hair bundle length and functional maturation of mammalian auditory hair cells. PLoS Biol. 9, e1001048 10.1371/journal.pbio.1001048 21526224 PMC3079587

[b44] Zhang M. Rao P. V. (2005). Blebbistatin, a novel inhibitor of myosin II ATPase activity, increases aqueous humor outflow facility in perfused enucleated porcine eyes. Invest. Ophthalmol. Vis. Sci. 46, 4130–4138 10.1167/iovs.05-0164 16249490

[b45] Zwaenepoel I. Naba A. Da Cunha M. M. Del Maestro L. Formstecher E. Louvard D. Arpin M. (2012). Ezrin regulates microvillus morphogenesis by promoting distinct activities of Eps8 proteins. Mol. Biol. Cell 23, 1080–1095 10.1091/mbc.E11-07-0588 22262457 PMC3302735

