# Supplementary material for: Eps8 controls Src- and FAK-dependent phenotypes in squamous carcinoma cells
Source: J Cell Sci. 2014 Dec 15;127(24):5303–16. doi: 10.1242/jcs.157560 (PMC4265741; doi:10.1242/jcs.157560)
Supplement: Supplementary Material [file supp_127_24_5303__index.html]

Eps8 controls Src- and FAK-dependent phenotypes in squamous carcinoma cells — Supplementary Material 

# Eps8 controls Src- and FAK-dependent phenotypes in squamous carcinoma cells

## JCS157560 Supplementary Material

**Files in this Data Supplement:**

- **Supplementary Material**
