# Supplementary material for: Eps8 controls Src- and FAK-dependent phenotypes in squamous carcinoma cells
Source: J Cell Sci. 2014 Dec 15;127(24):5303–16. doi: 10.1242/jcs.157560 (PMC4265741; doi:10.1242/jcs.157560)

SUPPLEMENTARY FIGURE LEGENDS

Figure S1: Eps8 is localized at focal adhesions and binds directly to FAK

(A) Focal adhesions were isolated from FAK WT and FAK -/- cells using hydrodynamic force. Focal adhesions (solid arrows) were stained with anti-Paxillin pY118 and anti-Eps8. Scale bars: 20 µm. **(B)** SCC FAK WT and -/- cells were treated with 50 µM blebbistatin for 1 h and then fixed and stained with anti-FAK and anti-Eps8 (left panel) or anti-Src pY416 and anti-Eps8 (right panel). Quantification is representative of three independent experiments. Scale bars: 20 µm. Error bars: s.d. $p < 0.001$. SCC FAK WT and -/- cells were treated with 50 µM blebbistatin for 1 h and then fixed and stained with **(C)** anti-FAK and anti-Eps8, **(D)** anti-Src pY416 and anti-Eps8 or **(E)** anti-Paxillin pY118 and anti-Eps8. Colocalization (COSTES r value of five cells) was analyzed using the ImageJ plugin JaCoP (Bolte and Cordelieres, 2006). Scale bars: 10 µm (unless otherwise stated).

Figure S2: Eps8 is not involved in SCC cell proliferation and random migration, but in cancer related processes mediated by FAK

(A) FAK WT and FAK -/- cells were transiently transfected with Eps8 siRNA or **(B)** infected with Eps8 (shE) or non-targeting (shC) shRNA to stably reduce Eps8 protein expression. Cell proliferation was analyzed by trypan blue staining and cells were counted using the Countess automated cell counter (Life Technologies, Paisley, UK). For random cell migration, SCC FAK WT and -/- cells transiently transfected with Eps8 siRNA were plated sparsely on plastic and random cell migration was recorded over 16 h. **(C)** Cell velocity, **(D)** track length and **(E)** track distance of 30 cells per experiment were analyzed using ImageJ. All experiments were carried out three times. FAK WT and FAK -/- cells were infected with Eps8 (shE) or non-targeting (shC) shRNA to stably reduce Eps8 protein expression. **(F)** Cells were plated on fibronectin coated coverslips. The confluent cell monolayer was wounded and subsequently fixed and stained with TRITC-phalloidin and anti-GM130. The experiment was repeated at least three times. Solid arrows: polarized cells. Dashed arrows: unpolarized cells. Scale bars: 20 µm. Error bars: s.d. $p < 0.005$. **(G)** Cells were seeded on growth factor reduced Matrigel. Invasion towards serum gradient > 80 µm was analyzed after three days. Error bars: s.e.m. $p < 0.001$. **(H)** FAK WT and FAK -/- cells with stable knockdown of Eps8 expression were seeded on fibronectin.

The cell monolayer was wounded and wound closure was analyzed after 15 h. Error bars: s.e.m. $p < 0.01$. All experiments were carried out at least three times.

Figure S3: c-Src-mediated Eps8 tyrosine phosphorylation correlates with Eps8 localization in SCC FAK -/- cells and Eps8 knockdown reduces the number of Src pY416 positive puncta in FAK -/- SCC cells

(A) SCC FAK WT and FAK -/- stably infected with Eps8 (shE) or non-targeting shRNA (shC) were treated with 100 nM Dasatinib for 30 min. Cells were lysed and Eps8 was immunoprecipitated using anti-Eps8 antibody, followed by western blotting analysis with the indicated antibodies. Immunoblotting with β-actin served as a loading control. **(B)** FAK -/- cells were grown on coverslips and treated with 100 nM Dasatinib for 24 h. Cells were fixed and stained with anti-Src and anti-Eps8 (left hand panels) or anti-LC3B and anti-Eps8 (right hand panels). Solid arrows indicate peripheral c-Src or Eps8 staining. Dashed arrows indicate internalized c-Src or Eps8 staining. Quantitation is representative of three independent experiments. Scale bars: 20 μm. Error bars: s.d. $p < 0.001$. Inhibition of active c-Src with 100 nM Dasatinib for 24 h was confirmed by western blotting analysis with the indicated antibodies. **(C)** FAK WT and FAK-/- cells were transfected with Eps8 siRNA, fixed 48 h post transfection and stained with anti-paxillin and anti-Src pY416. Results show typical staining observed across multiple experiments. Solid arrows indicate focal adhesions. Dashed arrows indicate internalized active c-Src. Scale bars: 20 μm. FAK WT and FAK -/- cells were infected with Eps8 (shE) or non-targeting (shC) shRNA to stably reduce Eps8 protein expression. Cells were grown on glass coverslips, fixed and stained with **(D)** anti-Src pY416 and anti-Eps8 or **(E)** anti-Src pY416 and anti-paxillin. Results show typical staining observed across multiple experiments. Solid arrows indicate focal adhesions. Dashed arrows indicate internalized active c-Src in autophagosomes. Scale bars: 20 μm. Error bars: s.d. $p < 0.001$.

Figure S4: Eps8 is not involved in general autophagy and knockdown of Eps8 results in loss of actin changes which are not associated with general autophagy

FAK WT and FAK -/- cells were transiently transfected with siEps8 and treated with 10 μM chloroquine for 24 h. **(A)** Cells were lysed and samples were subjected to western blot analysis using the indicated antibodies. **(B)** FAK -/- cells, transiently transfected with siEps8 and treated with chloroquine, were fixed and stained with

anti-LC3B and anti-Src pY416 (left panel) or anti-LC3B and anti-Eps8 (right panel). Scale bars: 20 μm. **(C)** SCC FAK -/- cells were transiently transfected with siEps8, fixed and stained with anti-Eps8 and TRITC-phalloidin (left panel) and anti-LC3B and TRITC-phalloidin (right panel). Scale bars: 20 μm.

Supplementary Figure S1

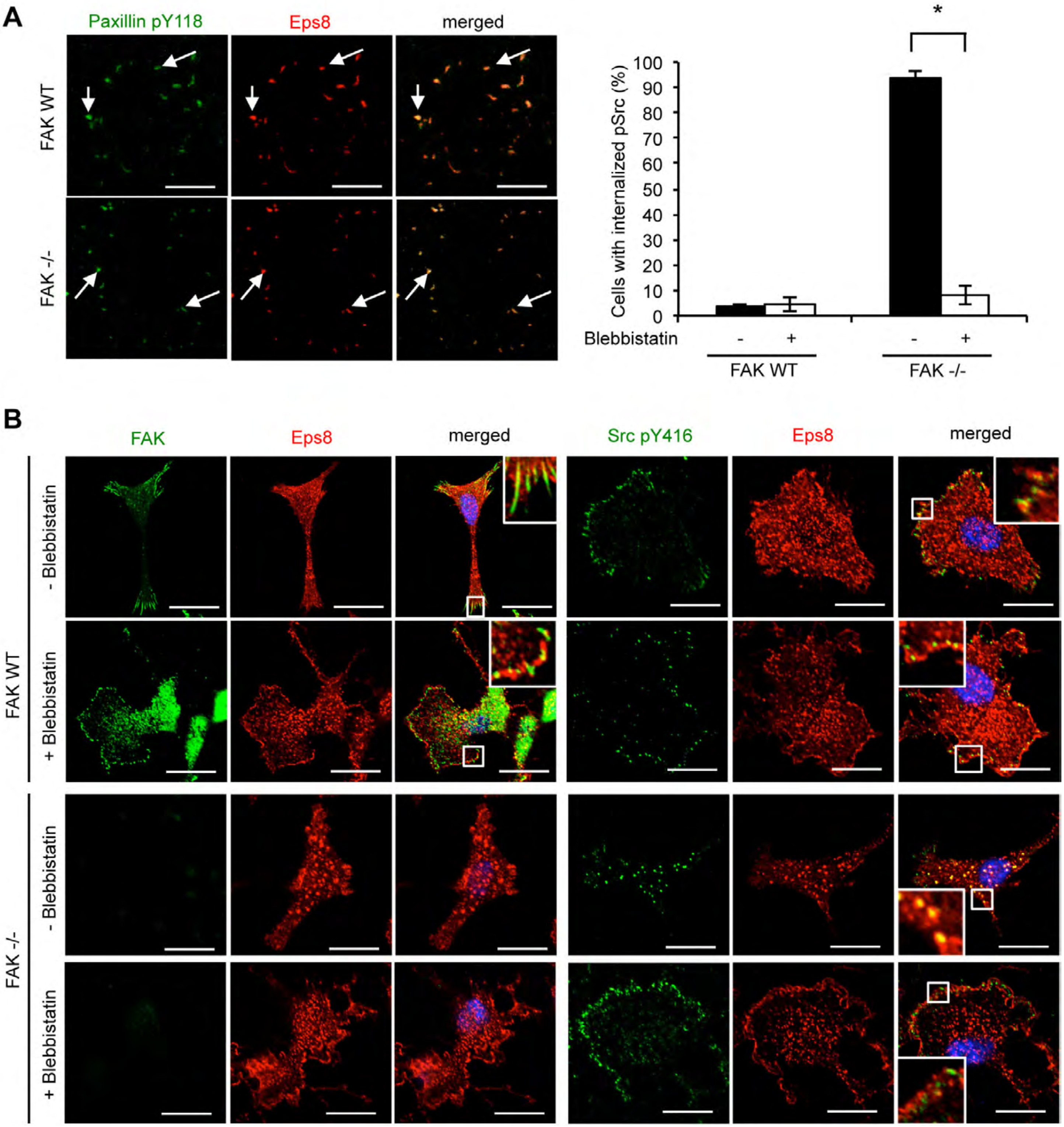

Supplementary Figure S1 continued

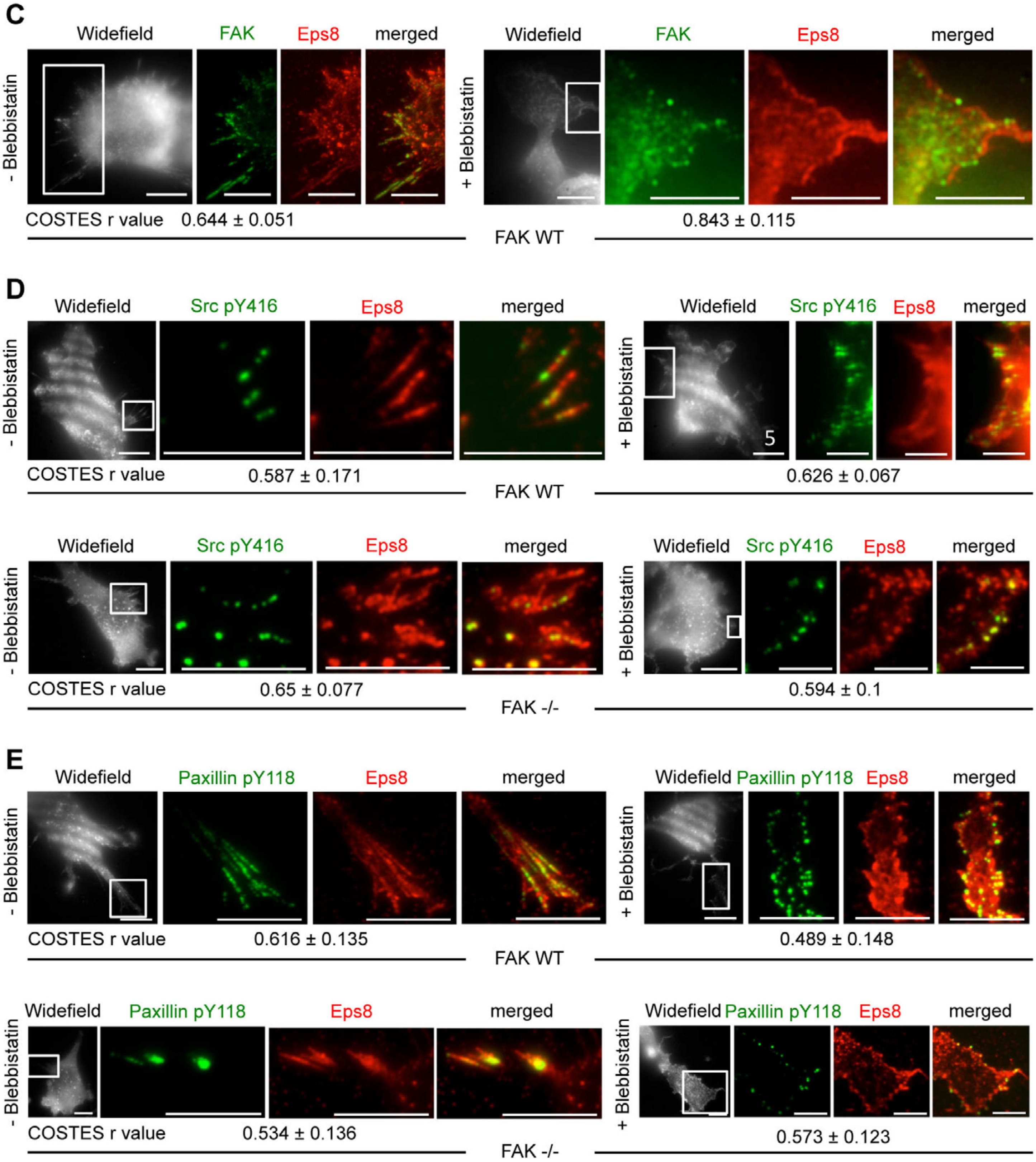

Supplementary Figure S2

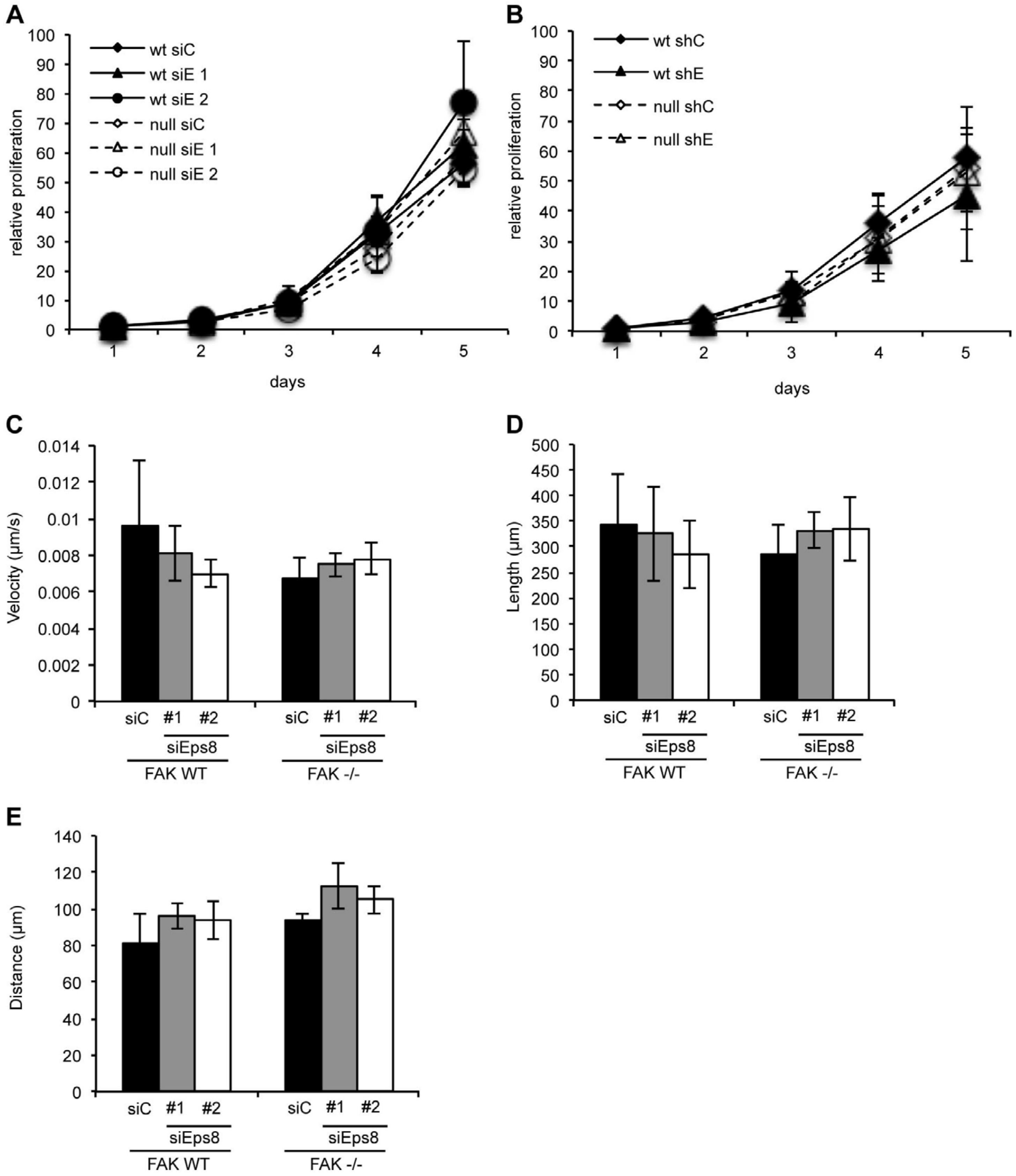

Supplementary Figure S2 continued

F

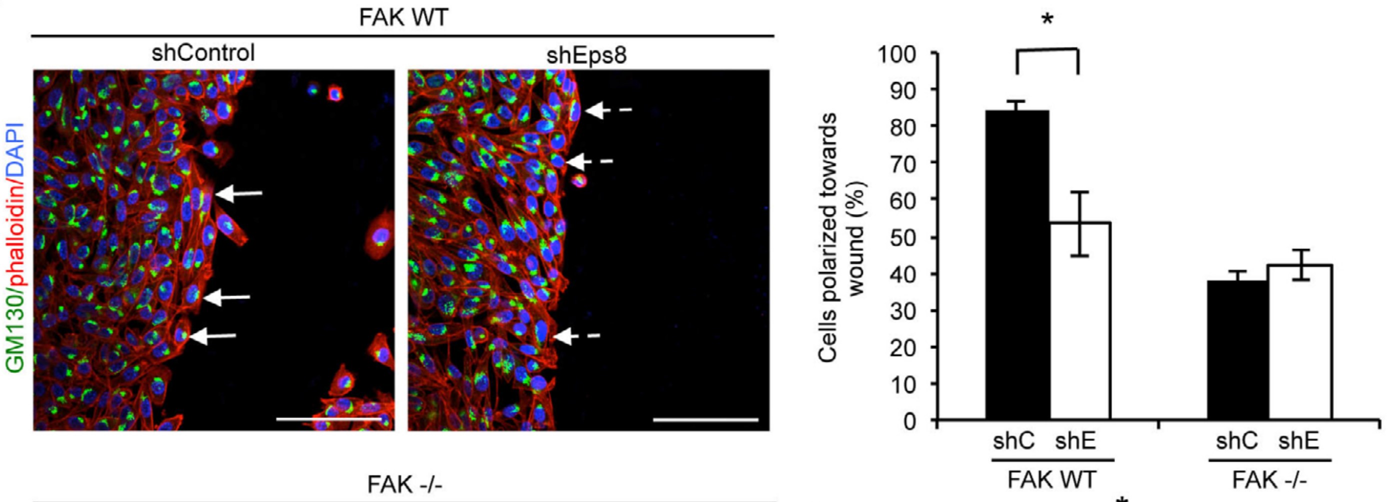

G

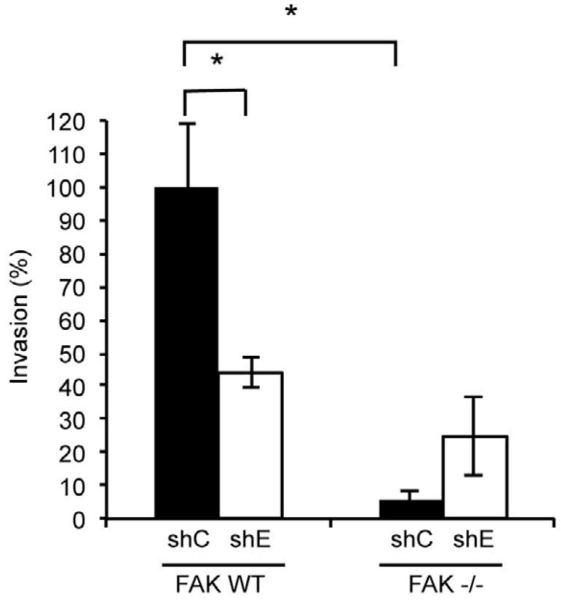

H

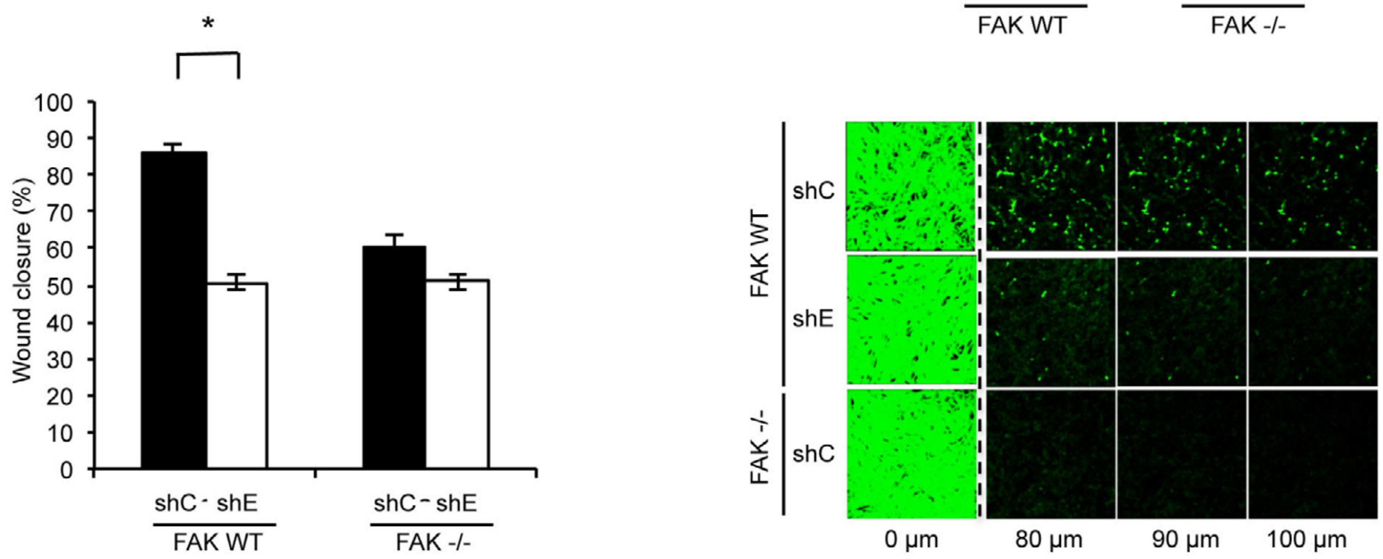

Supplementary Figure S3

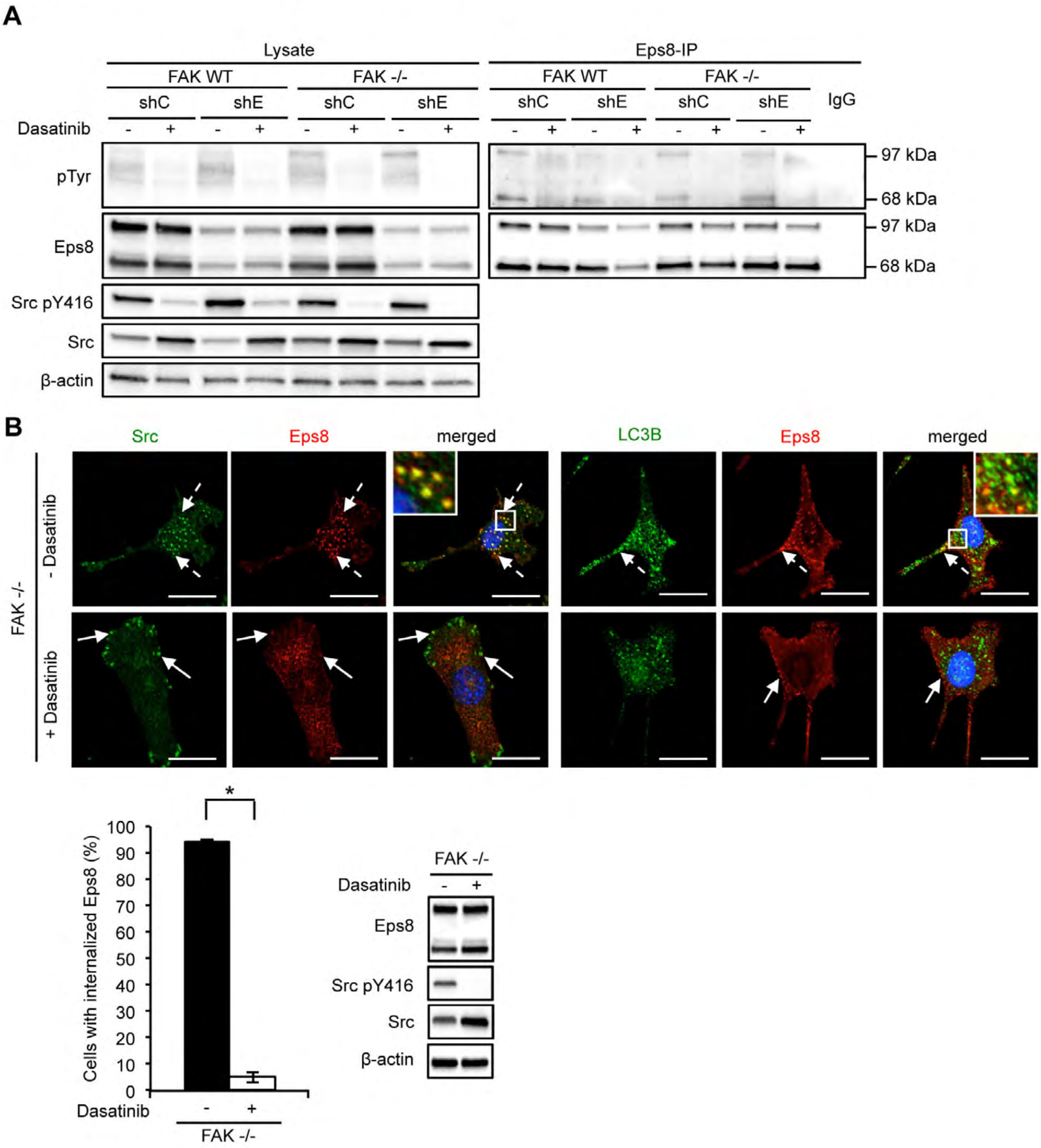

Supplementary Figure S3 continued

C

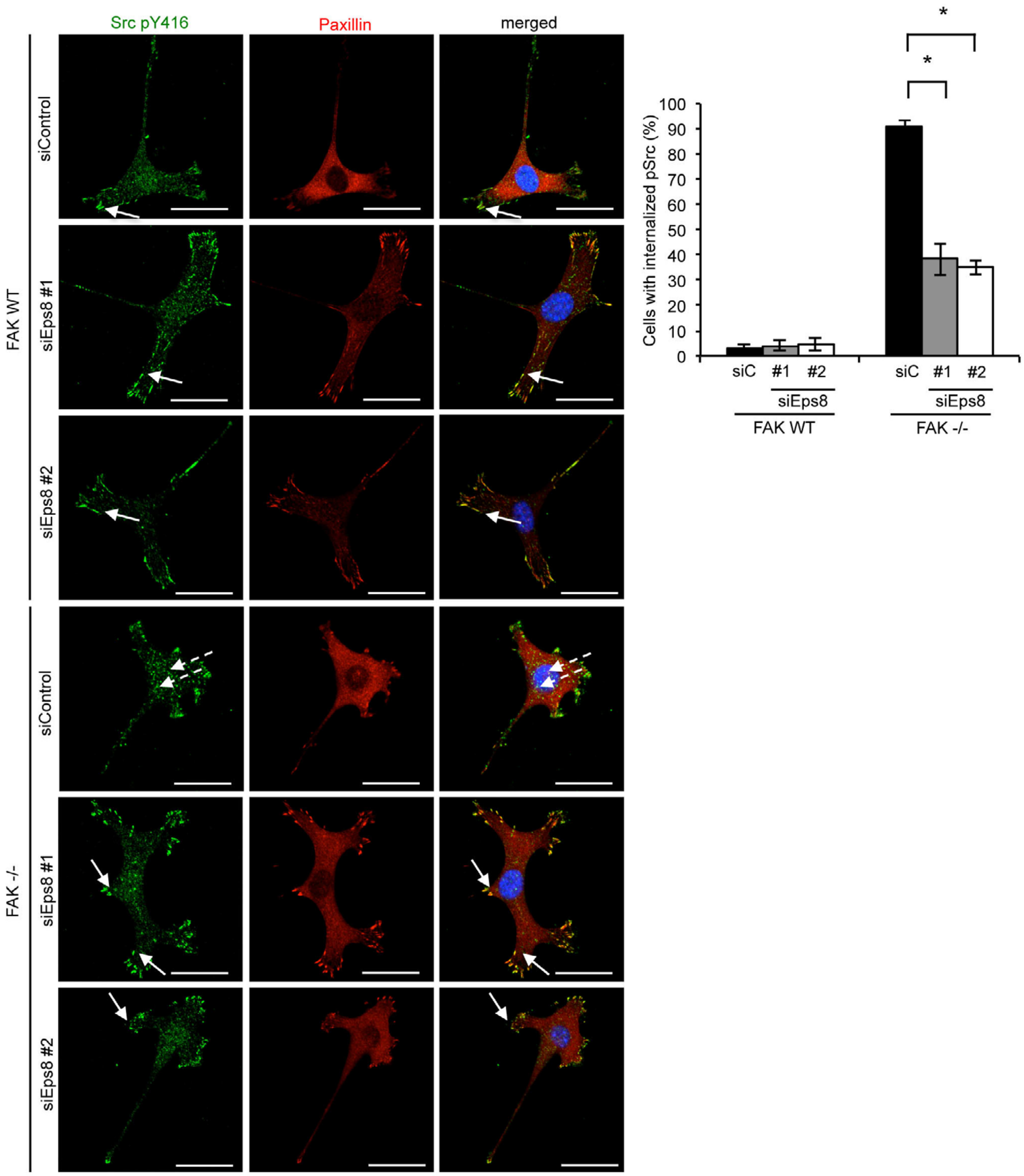

Supplementary Figure S3 continued

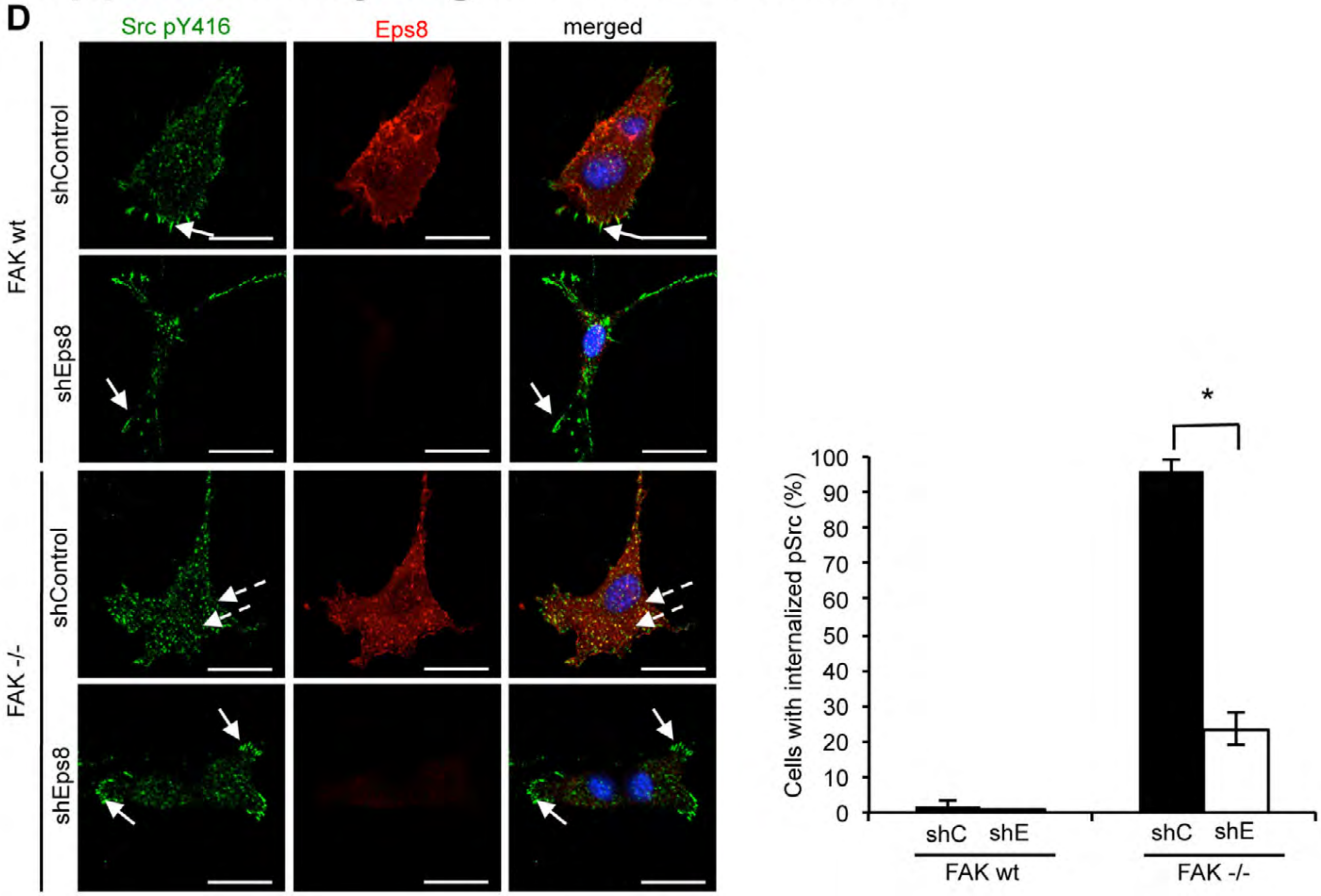

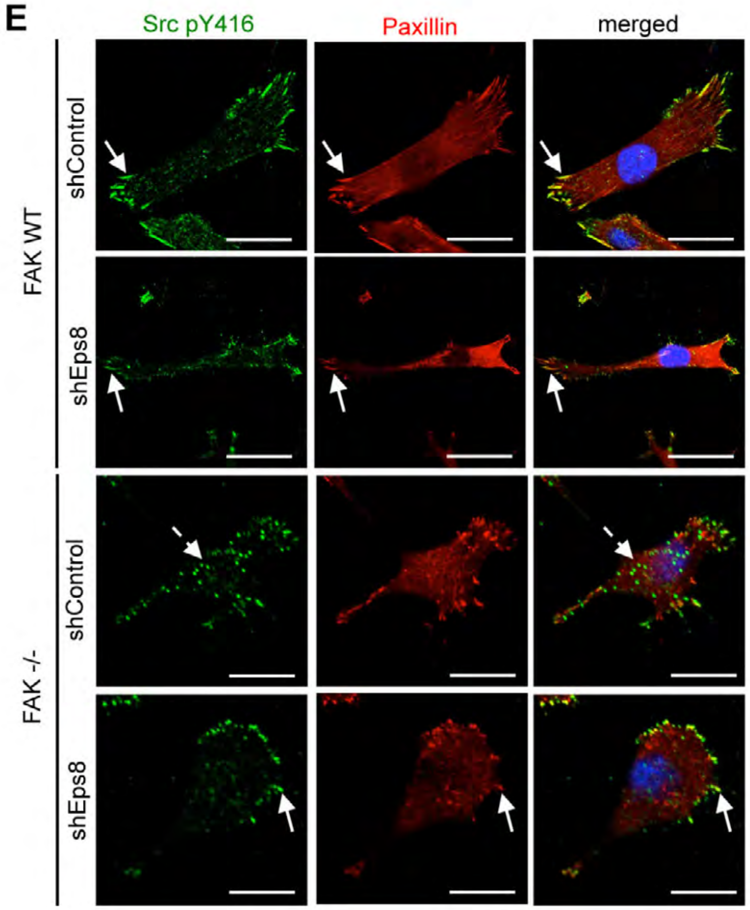

Supplementary Figure S4

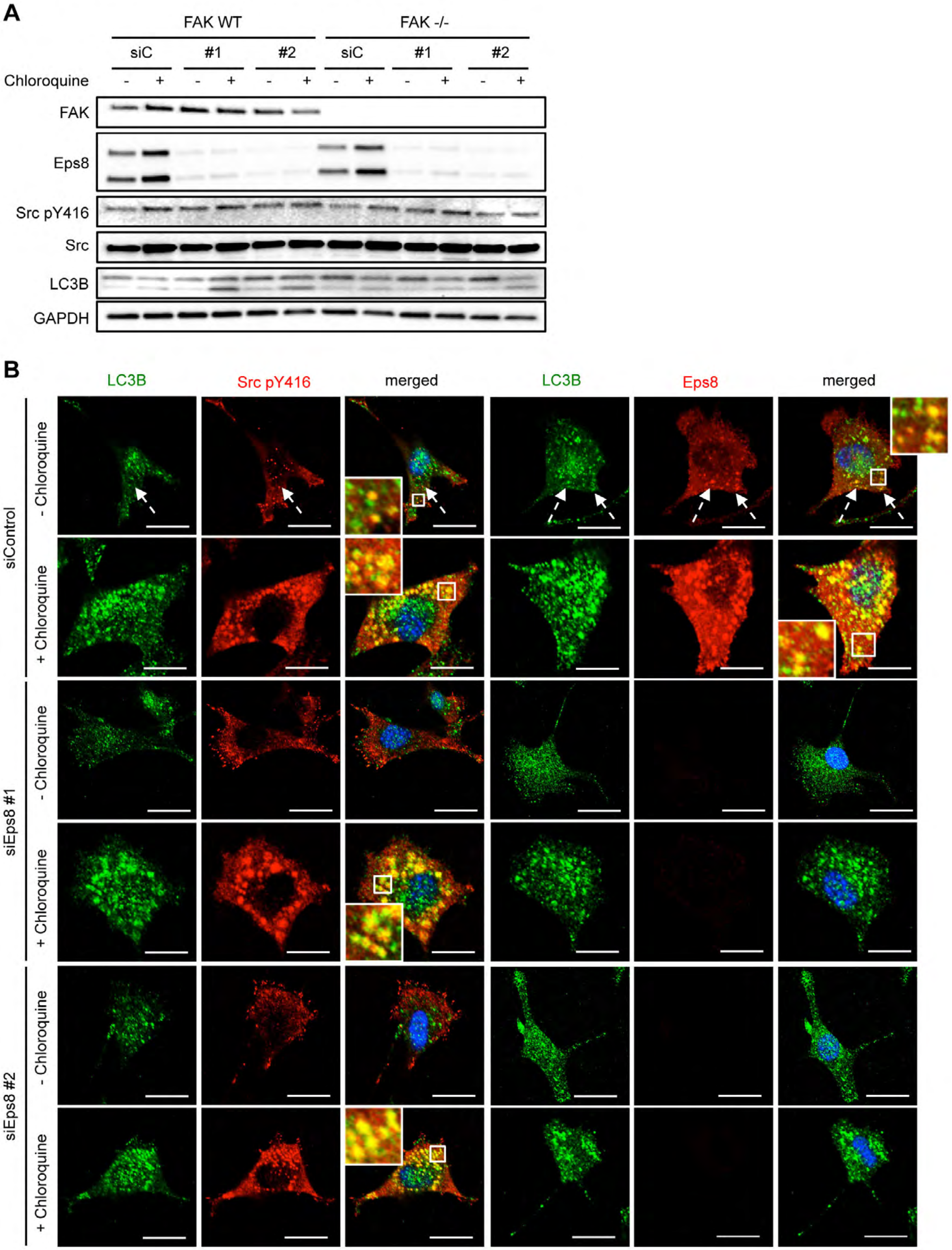

Supplementary Figure S4 continued

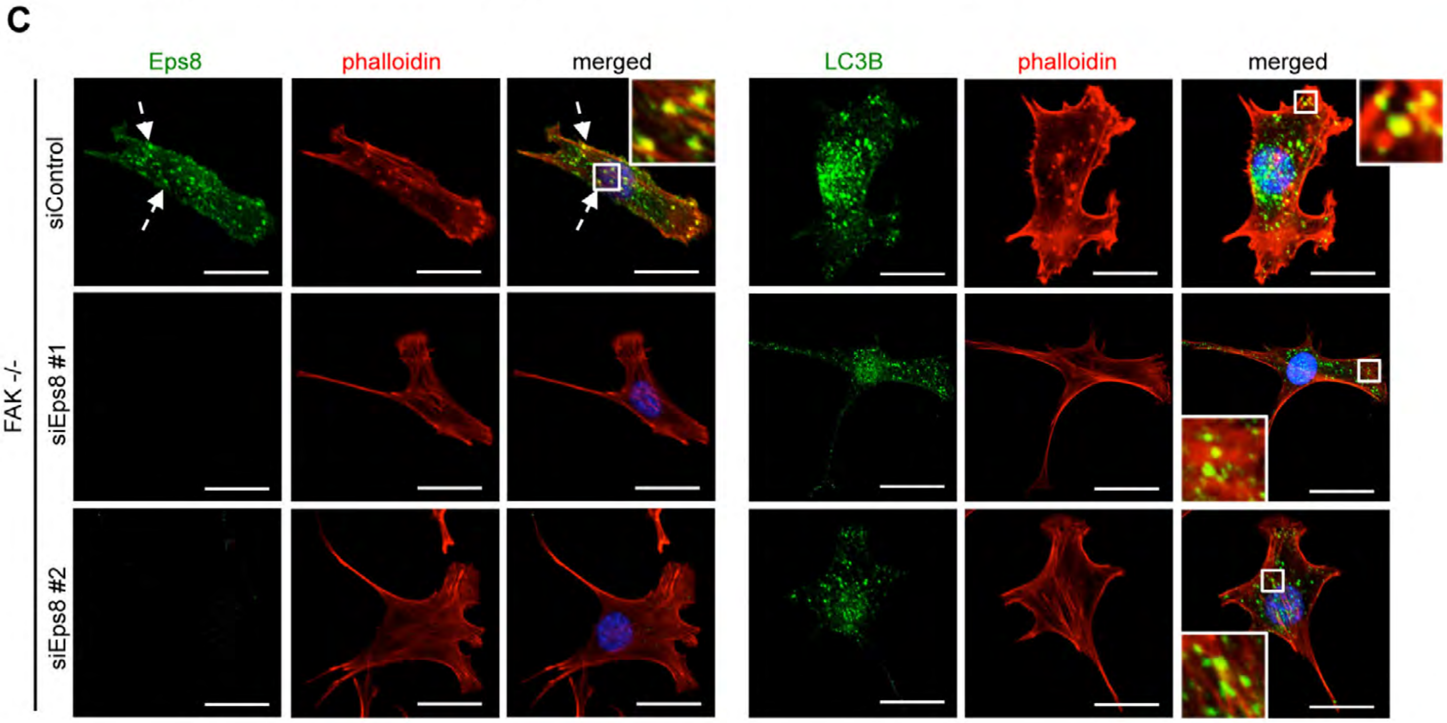

Supplement: Supplementary Material [file supp_127.24.5303_JCS157560.pdf]
